# Brain Region-Specific Expression of MeCP2 Isoforms Correlates with DNA Methylation within *Mecp2* Regulatory Elements

**DOI:** 10.1371/journal.pone.0090645

**Published:** 2014-03-03

**Authors:** Carl O. Olson, Robby M. Zachariah, Chinelo D. Ezeonwuka, Vichithra R. B. Liyanage, Mojgan Rastegar

**Affiliations:** Regenerative Medicine Program, and Department of Biochemistry and Medical Genetics, Faculty of Medicine, University of Manitoba, Winnipeg, Manitoba, Canada; University of Insubria, Italy

## Abstract

MeCP2 is a critical epigenetic regulator in brain and its abnormal expression or compromised function leads to a spectrum of neurological disorders including Rett Syndrome and autism. Altered expression of the two MeCP2 isoforms, MeCP2E1 and MeCP2E2 has been implicated in neurological complications. However, expression, regulation and functions of the two isoforms are largely uncharacterized. Previously, we showed the role of MeCP2E1 in neuronal maturation and reported MeCP2E1 as the major protein isoform in the adult mouse brain, embryonic neurons and astrocytes. Recently, we showed that DNA methylation at the regulatory elements (REs) within the *Mecp2* promoter and intron 1 impact the expression of *Mecp2* isoforms in differentiating neural stem cells. This current study is aimed for a comparative analysis of temporal, regional and cell type-specific expression of MeCP2 isoforms in the developing and adult mouse brain. MeCP2E2 displayed a later expression onset than MeCP2E1 during mouse brain development. In the adult female and male brain hippocampus, both MeCP2 isoforms were detected in neurons, astrocytes and oligodendrocytes. Furthermore, MeCP2E1 expression was relatively uniform in different brain regions (olfactory bulb, striatum, cortex, hippocampus, thalamus, brainstem and cerebellum), whereas MeCP2E2 showed differential enrichment in these brain regions. Both MeCP2 isoforms showed relatively similar distribution in these brain regions, except for cerebellum. Lastly, a preferential correlation was observed between DNA methylation at specific CpG dinucleotides within the REs and *Mecp2* isoform-specific expression in these brain regions. Taken together, we show that MeCP2 isoforms display differential expression patterns during brain development and in adult mouse brain regions. DNA methylation patterns at the *Mecp2* REs may impact this differential expression of *Mecp2*/MeCP2 isoforms in brain regions. Our results significantly contribute towards characterizing the expression profiles of *Mecp2*/MeCP2 isoforms and thereby provide insights on the potential role of MeCP2 isoforms in the developing and adult brain.

## Introduction

Mutation or altered expression of the X-linked Methyl CpG Binding Protein 2 (*MECP2*) gene leads to a wide spectrum of neurodevelopmental disorders including autism spectrum disorders and Rett Syndrome [1]–[3]. Recent reports also show the role of MeCP2 in alcoholism and Fetal Alcohol Spectrum Disorders (FASD) [4]–[7]. MeCP2 is a multifunctional epigenetic factor that is involved in multiple nuclear events including transcriptional repression, transcriptional activation, RNA splicing, and chromatin compaction [8], [9]. MeCP2 was first discovered as a repressor protein that binds to methylated DNA at the 5-methylcytosine (5 mC) residues [10]. However, recent studies have shown that MeCP2 also binds to 5-hydroxymethylcytosine (5 hmC), presumably associated with transcriptional activators [11], [12]. While 5 mC is a hallmark of inactive genes [9], 5 hmC is generally associated with active genes [11]. Currently, it is unclear how, as a single protein MeCP2 provides so many different nuclear functions. One possible explanation could be the existence of redundant and non-redundant functions between the two MeCP2 isoforms.

In mice and humans, alternative splicing of the *Mecp2/MECP2* gene leads to the generation of two protein isoforms, MeCP2E1 and MeCP2E2 [13], [14]. MeCP2E1 contains a unique 21 amino acid sequence at its N-terminus, whereas the N-terminus of MeCP2E2 includes 9 exclusive amino acids [13], [14]. Except for their N-terminal regions, MeCP2 isoforms are similar and share the same functional domains, including the Methyl Binding Domain (MBD), and the Transcriptional Repression Domain (TRD) [2]. However, several previous studies indicate differential properties of MeCP2E1 and MeCP2E2 regarding their interacting protein partners, impact on neuronal survival [15], role during embryonic development [16],_ENREF_14 and sensitivity to different drugs such as Decitabine [17]. Moreover, both MeCP2 isoforms are involved in neurite formation [18], [19]. The majority of the research work on MeCP2 isoforms is focused on MeCP2E1, since it is considered to be the major isoform in the brain [20]–[22]. However, independent research groups have implicated significance of both MeCP2 isoforms in neurological/neurodevelopmental disorders. For instance, MeCP2E1 is considered as the most relevant isoform in RTT pathology [22], however several studies have shown altered expression of both *MECP2E1* and *MECP2E2* and disruption of *MECP2* alternative splicing in RTT patients with or without *MECP2* mutations [23]–[25]. Both MeCP2 isoforms can rescue RTT phenotypes in mice to different extents [26]. All these reports suggest that both MeCP2 isoforms are important in maintaining normal brain function and altered expression of both isoforms may lead to neurological complications. These reports also highlight the significance of understanding the expression, regulation and function of both MeCP2 isoforms in brain. Therefore, future directions should be aimed at elucidating the relevance of individual MeCP2 isoforms in mammalian neurophysiology.

The knowledge on the expression profiles of *MECP2/Mecp2* isoforms were limited to the transcript levels [14], [20], until 2012 when we reported MeCP2E1 distribution in the adult mouse brain, as well as embryonic cortical neurons and astrocytes [21]. Due to the lack of anti-MeCP2E2 antibodies, comparative analysis of both MeCP2 isoforms at the protein levels in any system has not been reported to date. The current study begins to address the need for comparative analysis of the expression of both *Mecp2*/MeCP2 isoforms in the developing and adult mouse brain.

Although previous studies highlight the importance of proper regulation of both *MECP2/Mecp2*/MeCP2 isoforms in the brain, the regulatory mechanisms of these isoforms still remain to be understood. It is known that *MECP2/Mecp2* expression is controlled by regulatory elements (REs) within the *MECP2/Mecp2* promoter and intron 1 [27]–[29]. Promoter DNA methylation is associated with altered *MECP2* expression in autistic brains [30], [31], and altered *Mecp2* expression in the brains of stressed mice [32]. Recently, we demonstrated that DNA methylation at the REs within the *Mecp2* promoter and intron 1 impact the expression of *Mecp2* isoforms in differentiating brain-derived neural stem cells (NSCs) [17]. In this current study, we detected differential expression of MeCP2 isoforms in a brain region-specific manner; therefore we investigated whether DNA methylation at the same REs correlates with the expression of *Mecp2* isoforms in the corresponding adult mouse brain regions.

In this report, we demonstrate differential expression profiles of MeCP2 isoforms during mouse brain development and in different brain regions of the adult mouse using our newly generated anti-MeCP2E1 and anti-MeCP2E2 antibodies. Using confocal microscopy, we show the detection of MeCP2 isoforms in the three main cell types of male and female mouse brain hippocampus, namely neurons, astrocytes, and oligodendrocytes. Furthermore, we show the differential distribution of MeCP2E1 and MeCP2E2 at different cellular layers of the adult male cerebellum. We performed bisulfite pyrosequencing at the *Mecp2* REs to show that differential DNA methylation at specific *Mecp2* REs are associated with *Mecp2e1* and *Mecp2e2* expression in the adult mouse brain regions. To our knowledge, this is the first report to compare *Mecp2*/MeCP2 isoforms at the transcript and protein levels in correlation with DNA methylation at the genomic level. Our results provide significant insight to the contribution of individual MeCP2 isoforms in mammalian brain development and function, as well as the potential impact of DNA methylation on *Mecp2* isoforms.

## Results

### MeCP2E2 expression displays a later onset than MeCP2E1 during mouse brain development

**Figure 1:**
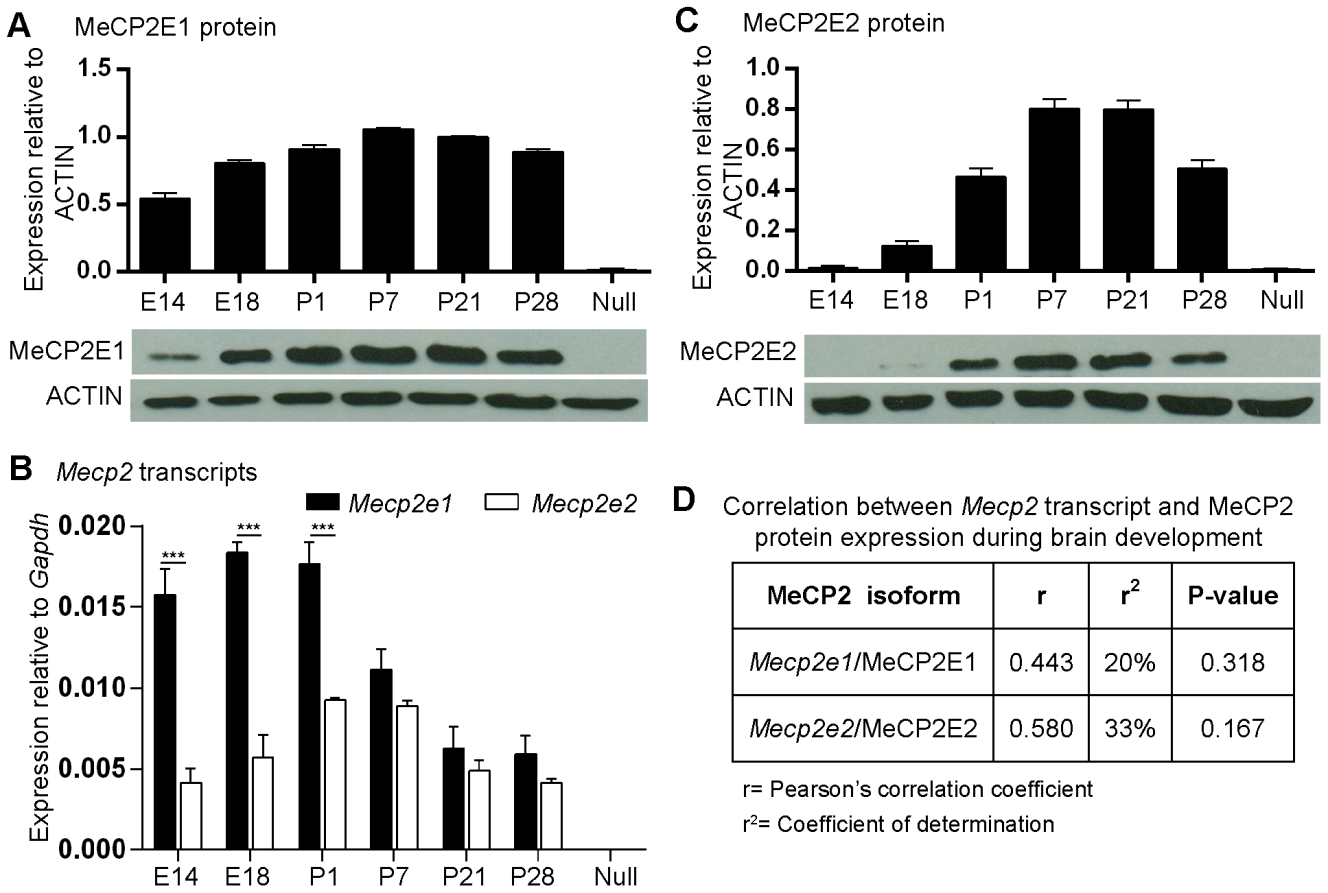
Transcript and protein expression of MeCP2 isoforms during mouse brain development. **(A)** Western blot analysis of MeCP2E1 in the isolated nuclear extracts from the whole brain at the indicated developmental time points. ACTIN was used as a loading control. N = 3±SEM. **(B)** Quantitative RT-PCR with specific primers to detect *Mecp2e1* and *Mecp2e2* transcripts. N = 3±SEM. Significant differences are indicated with P<0.001***. **(C)** Similar to A for MeCP2E2. N = 3±SEM. For A and C, significant differences in the levels of each MeCP2 isoform between different developmental time points are found in Table S5. **(D)** Pearson's correlation analysis for indicated *Mecp2* transcripts and MeCP2 protein levels. r =  Pearson's correlation coefficient, r^2^ = coefficient of determination, P<0.05*. E: embryonic days, P: postnatal days, null: *Mecp2* ^tm1.1Bird y/−^ brain tissue (nuclear extracts used as negative controls for A and C, total RNA was used as negative control in B).

The temporal expression profile of MeCP2 has been shown to increase during the course of mouse brain development and follows neuronal maturation [33], [34]. Therefore, we first aimed to study MeCP2E1 and MeCP2E2 expression during brain development before and after birth. Our studies included brain tissues from embryonic day (E) 14, E18, postnatal day (P) 1, P7, P21, and P28. Expression of both *Mecp2*/MeCP2 isoforms at the transcript and protein levels was studied by qRT-PCR and Western blot (WB) experiments, respectively. To study MeCP2E1 expression during mouse brain development, we used our previously reported anti-MeCP2E1 antibody [21]. We prepared nuclear extracts from dissected whole brain tissues at the aforementioned developmental time points for WB experiments. As MeCP2 is reported to be a nuclear protein [10], we first confirmed the detection of MeCP2E1 specifically in the nuclear fractions of our samples. Therefore, we loaded increasing concentrations of brain nuclear and cytoplasmic extracts and subjected them to WB analysis with anti-MeCP2E1 antibody. As expected, MeCP2E1 was only detected in the nuclear fractions, and not in cytoplasmic fractions (Figure S1A), indicating that in the adult brain, MeCP2E1 is a nuclear protein. Further WB experiments using developmental brain samples indicated that MeCP2E1 is detected as early as E14. We observed a gradual increase in MeCP2E1 expression levels, which reached a plateau between P7 to P21, and subsequent decline at P28 that was not statistically significant (Figure 1A; Table S1). Surprisingly, *Mecp2e1* transcripts were significantly higher at E14 and E18, with noticeable decline after birth (Figure 1B; Table S1). In all our protein and transcript analysis studies, we used *Mecp2* null mouse brain (*Mecp2* ^tm1.1Bird y/−^) as a negative control, since this mouse model is reported to lack *Mecp2*/MeCP2 transcript and protein expression [21], [35]. Pearson's correlation analysis (r) was performed between MeCP2E1 protein and *Mecp2e1* transcript levels, and no significant relation was observed between *Mecp2e1*/MeCP2E1 at the analyzed time points (r = 0.44) (Figure 1D).

**Figure 2:**
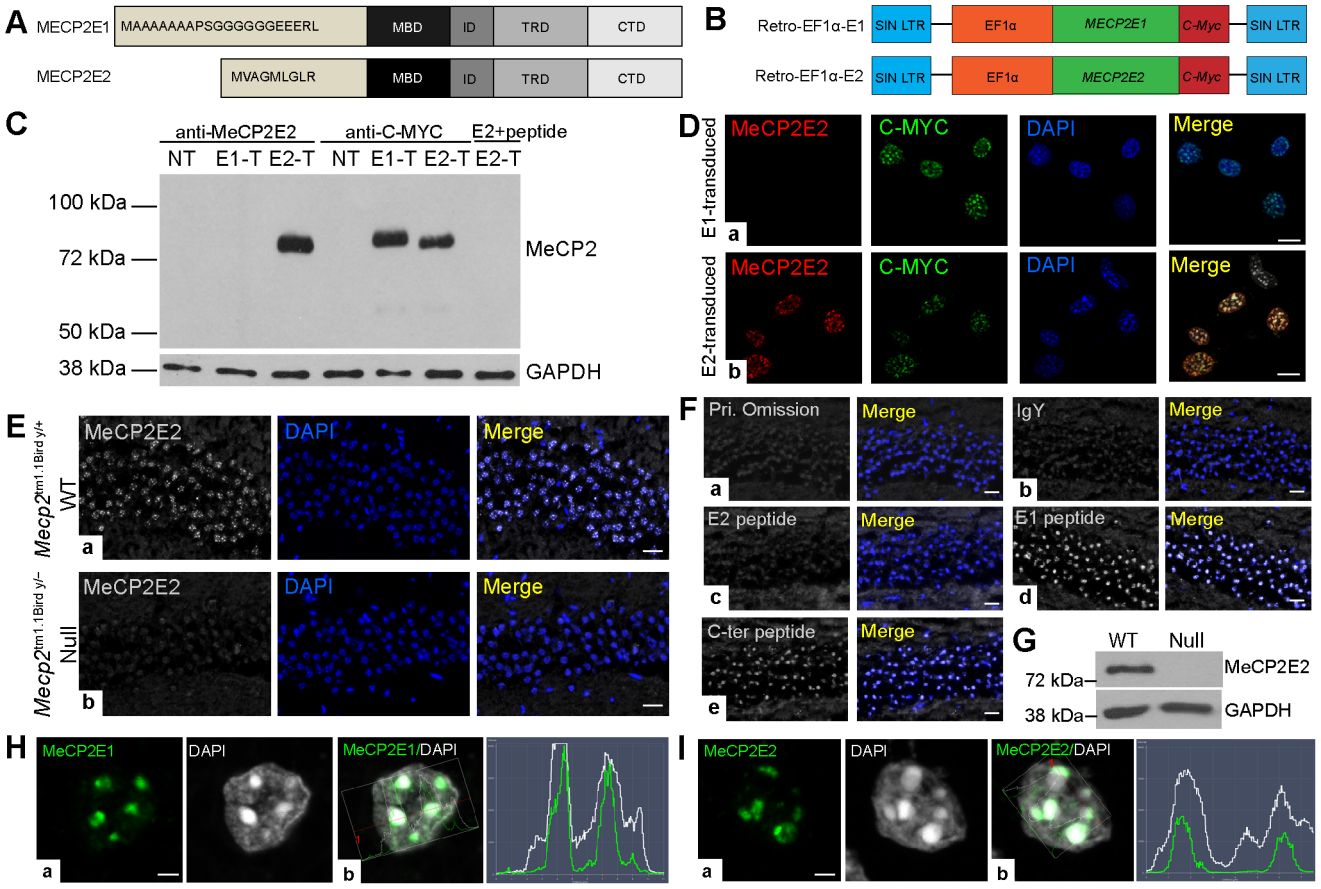
Validation of anti-MeCP2E2 antibody and detection of MeCP2 isoforms in mouse brain. **(A)** Schematic representation of MeCP2E1 and MeCP2E2 protein structures, differing only in their N-terminal sequences. MBD: methyl binding domain, ID: intervening domain, TRD: transcriptional repression domain, CTD: C-terminal domain (adapted from [21]). **(B)** Schematics of *MECP2E1* (Retro-EF1α-E1) and *MECP2E2* (Retro-EF1α-E2) retroviral vectors with C-MYC tag that were used for transfection of Phoenix cells (in C) and transduction of NIH3T3 cells (in D) (adapted from [18]). **(C)** Western blot (WB) experiment to detect MeCP2E2 expression in control non-transfected (NT), *MECP2E1* transfected (E1-T), *MECP2E2* transfected (E2-T), and E2-T pre-incubated with E2 antigenic peptide. Anti-MYC labelling was used as a positive control. **(D)** MeCP2E2 detection by immunofluorescence staining in transduced NIH3T3 cells with either a) *MECP2E1*, or b) *MECP2E2* retroviral vectors. **(E)** Detection of endogenous MeCP2E2 by immunohistochemistry in the CA1 region of adult mouse hippocampus from a) wild type (WT) (*Mecp2* ^tm1.1Bird y/+^), and b) null (*Mecp2* ^tm1.1Bird y/−^) *Mecp2* mice. **(F)** Controls to verify the specificity of anti-MeCP2E2 by IHC in the adult mouse brain; a) primary omission, b) anti-MeCP2E2 incubation with IgY, pre-incubation of the newly generated anti-MeCP2E2 antibody with the antigenic peptide against c) MeCP2E2, d) MeCP2E1, e) C-terminus of MeCP2. **(G)** Western blot to detect MeCP2E2 in the WT adult mouse brain and *Mecp2* null mice. GAPDH was used as a loading control. **(H)** a) Confocal images of MeCP2E1 in WT adult mouse brain hippocampus CA1 region. b) Signal intensity profile analysis indicates the enrichment of MeCP2E1 at the DAPI-rich heterochromatin regions of nuclei. **(I)** a) Confocal images of MeCP2E2 in WT adult mouse brain hippocampus CA1 region. b) Signal intensity profile analysis of MeCP2E1 and DAPI co-localization indicating MeCP2E2 detection at the DAPI-rich heterochromatin regions of nuclei. Scale bars represent 20 µm in D–E, 10 µm in D, and 2 µm in H–I.

Next, we performed a similar analysis of *Mecp2e2*/MeCP2E2 expression at these selected developmental time points. As there was no commercially available anti-MeCP2E2-specific antibody, we generated and validated a novel chicken polyclonal anti-MeCP2E2 antibody using an antigenic peptide corresponding to the N-terminus region of MeCP2E2, with similar approach that we previously reported for MeCP2E1 [21]. We validated the specificity of this novel anti-MeCP2E2 antibody by multiple techniques including WB and immunofluorescence (IF) in transfected Phoenix cells (for WB) and transduced NIH3T3 cells (for IF) with Retro-EF1α-E1 or Retro-EF1α-E2 [18] (Figure 2B). Control studies included non-transfected Phoenix and non-transduced NIH3T3 cells (Figure 2C-D; Figure S2). For detailed description of antibody validation in Phoenix and NIH3T3 cells, refer to Note S1. Our novel anti-MeCP2E2 antibody showed positive signals in WT adult mouse hippocampus CA1 region in brain by IHC experiments (Figure 2E-a). As expected, MeCP2E2 signals were not detected in the null mice brain (Figure 2E-b). Further control experiments showed that MeCP2E2 signals were eliminated by MeCP2E2-antigenic peptide, but not when the antibody was pre-incubated with MeCP2E1-specific or a MeCP2 C-terminal-specific peptide (Abcam) (Figure 2F). Additional controls with primary omission and IgY incubation did not result in any detectable signal, as expected (Figure 2F). By WB analysis with anti-MeCP2E2 antibody, we detected a clear band at ∼75kDa in WT brain nuclear extracts which was absent in the null mice brain (Figure 2G). The MeCP2E2 signal from the WT mouse brain was only present in the nuclear extracts and not cytoplasmic extracts (Figure S1B), indicating that similar to MeCP2 (total) and MeCP2E1, MeCP2E2 is also a nuclear protein. Within the nucleus, we have reported that MeCP2E1 is enriched at the DAPI-rich chromocenters [21]. Similar to MeCP2E1 (Figure 2H), confocal microscopic analysis of a single nucleus from adult mouse hippocampus showed that MeCP2E2 is also enriched in the DAPI-rich chromocenters (Figure 2I).

After confirming that the newly developed anti-MeCP2E2 antibody specifically detects endogenous MeCP2E2, we investigated MeCP2E2 expression levels during mouse brain development. Western blot analysis of nuclear extracts from selected developmental time points indicated that MeCP2E2 has a delayed onset of protein expression compared to MeCP2E1, with the earliest detection at E18 (Figure 1C). From E18 onwards, MeCP2E2 showed a similar expression pattern when compared to MeCP2E1, albeit at lower levels (Figure 1C; Table S1). On the other hand, *Mecp2e2* transcript levels increased prenatally until birth with highest levels at P1-P7, and significantly declined by P28 (Figure 1B; Table S1). Comparison of *Mecp2e1*/*Mecp2e2* transcript levels indicated significantly higher *Mecp2e1* levels from E14 until birth; however no significant difference in transcript expression was detected between P7-P28 (Figure 1B). Pearson's correlation analysis showed no significant relation between *Mecp2e2*/MeCP2E2 transcript and protein (r = 0.58) at these selected developmental time points (Figure 1D). In order to confirm the detection of MeCP2 expression during murine brain development, we also performed WB experiments using a commercially available MeCP2 antibody (specific to C-terminus; referred to as total MeCP2), which detects both MeCP2 isoforms (Figure S3A). As expected, this antibody detected the total MeCP2 from E14 to E28, overlapping with all the time points that MeCP2E1 and/or MeCP2E2 are detected. No signal was detected with nuclear extracts from *Mecp2* null brain.

Taken together, our results confirm that in the adult mouse brain MeCP2E2 is a nuclear protein that is enriched in the chromocenters of the hippocampus CA1 nuclei. We show that while the onset of expression of MeCP2E1 and MeCP2E2 significantly differs prenatally during mouse brain development, their expression overlap after birth. Additionally, our studies indicate that there is no significant correlation between *Mecp2*/MeCP2 isoform-specific transcript and protein expression levels during mouse brain development.

### MeCP2 protein isoforms show similar cell type-specific patterns in brain hippocampus of both male and female adult mice

It has been reported that MeCP2 is expressed in the three main brain cell types including neurons, astrocytes and oligodendrocytes [17], [18], [21], [36], [37]. Accordingly, *Mecp2*-deficiency in these cell types is associated with neurological dysfunction [37], therefore indicating the importance of MeCP2 function in these cells. Since we detected consistent and uniform expression pattern for both MeCP2E1 [21] and MeCP2E2 (Figure 2E) in the nuclei of hippocampal cells in CA1 region; we further investigated MeCP2E1 and MeCP2E2 expression in neurons, astrocytes and oligodendrocytes in the hippocampus.

**Figure 3:**
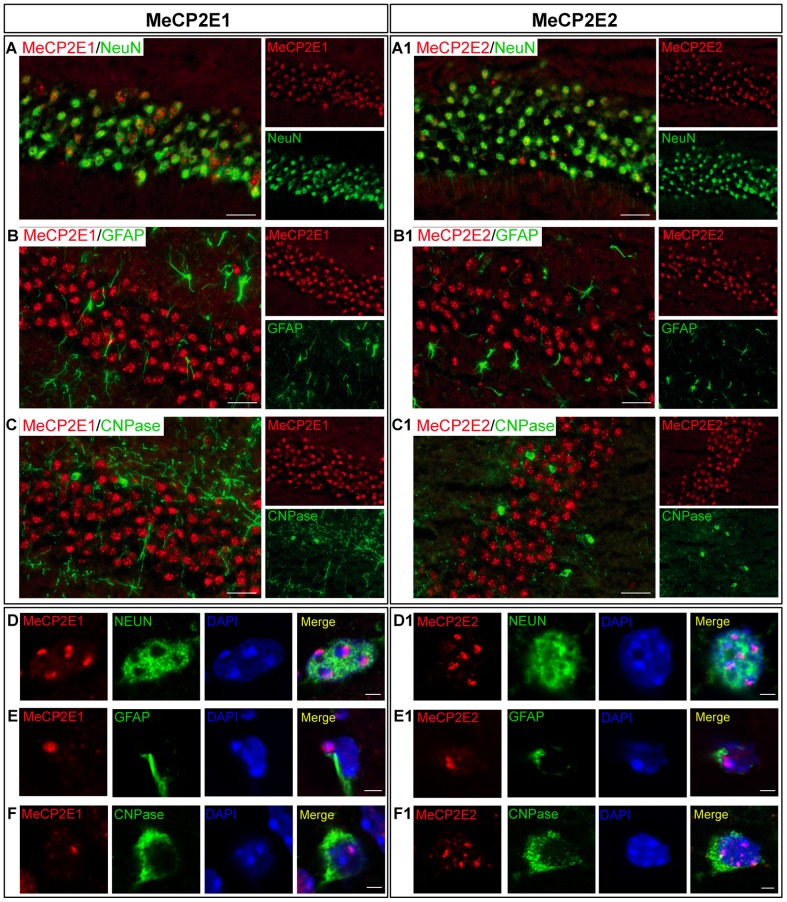
Detection of MeCP2E1 and MeCP2E2 in different cell types of the hippocampus (CA1 region) in adult male mouse brain. Left panel **(A**–**F)**: MeCP2E1 and, Right panel **(A1**–**F1)**: MeCP2E2. Expression of MeCP2 isoforms was analyzed in neurons (NEUN^+^), astrocytes (GFAP^+^), and oligodendrocytes (CNPase^+^). **(A**–**C)** and **(A1**–**C1)** represent ×40 images. Scale bars represent 20 µm. **(D**–**F)** and **(D1**–**F1)** represent confocal images of single nuclei. Scale bars represent 2 µm. **(A**–**A1)** and **(D**–**D1)**: Expression of MeCP2 isoforms in NEUN^+^ neurons. **(B**–**B1)** and **(E**–**E1)**: Expression of MeCP2 isoforms in GFAP^+^ astrocytes. **(C**–**C1)** and **(F**–**F1)**: Expression of MeCP2 isoforms in CNPase^+^ oligodendrocytes. All images were taken from hippocampus of adult male mouse brain.

In male mouse brain, immunofluorescence co-labelling of MeCP2E1 with a neuronal nuclei marker NeuN showed that the majority of MeCP2E1-labelling was localized to NeuN^+^ positive cells in the hippocampus CA1 layer (Figure 3A). Similar to MeCP2E1, we also detected MeCP2E2 present in the majority of neuronal nuclei in the hippocampus CA1 layer of male mice (Figure 3A1). Confocal analysis confirmed similar nuclear expression patterns for MeCP2E1 and MeCP2E2 in NeuN^+^ nuclei (Figure 3D-D1). We observed less frequent immunofluorescence detection of MeCP2E1 and MeCP2E2 in DAPI-counterstained NeuN^−^ cells, indicating the possibility for expression of MeCP2 isoforms in non-neuronal cells. Immunofluorescence co-labelling experiments with anti-MeCP2E1 or anti-MeCP2E2 antibodies in combination with an astrocyte marker (GFAP), and an oligodendrocyte marker (CNPase) indicated the presence of both MeCP2 isoforms in these cell types (Figure 3B-B1; C-C1). However, confocal microscopy imaging analysis suggested that the expression of both MeCP2 isoforms in astrocytes and oligodendrocytes were lower than their expression in neurons (Figure 3 E-E1; F-F1). This is in agreement with previous reports showing lower levels of MeCP2 in astrocytes and oligodendrocytes [37]. As expected, we did not detect any specific signal for either isoform in GFAP^+^ astrocytes and CNPase^+^ oligodendrocytes, when IHC experiments were repeated in the brain of adult null mice (Figure S4).

**Figure 4:**
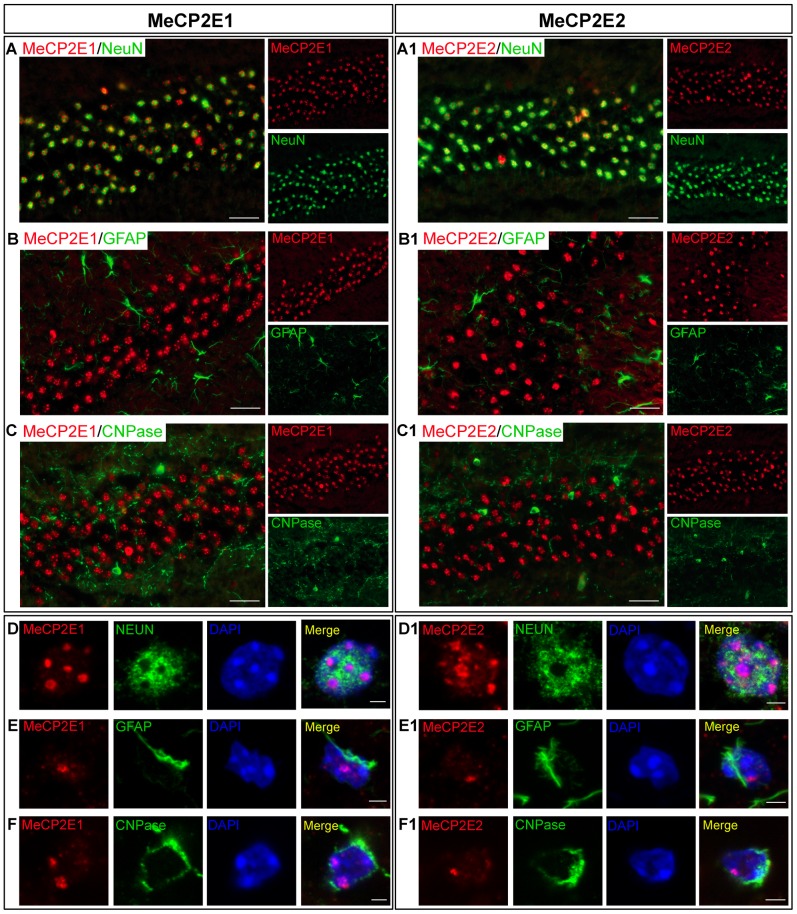
Detection of MeCP2E1 and MeCP2E2 in different cell types of the hippocampus (CA1 region) in adult female mouse brain. Left panel **(A**–**F)**: MeCP2E1 and, Right panel (**A1**–**F1)**: MeCP2E2. Expression of MeCP2 isoforms was analyzed in neurons (NEUN^+^), astrocytes (GFAP^+^), and oligodendrocytes (CNPase^+^). **(A**–**C)** and **(A1**–**C1)** represent ×40 images. Scale bars represent 20 µm. **(D**–**F)** and **(D1**–**F1)** represent confocal images of single nuclei. Scale bars represent 2 µm. **(A**–**A1)** and **(D**–**D1)**: Expression of MeCP2 isoforms in NEUN^+^ neurons. **(B**–**B1)** and **(E**–**E1)**: Expression of MeCP2 isoforms in GFAP^+^ astrocytes. **(C**–**C1)** and **(F**–**F1)**: Expression of MeCP2 isoforms in CNPase^+^ oligodendrocytes. All images were taken from hippocampus of adult female mouse brain.

In the female mouse brain, we detected immunofluorescence -labelling similar to that of male for MeCP2E1 and MeCP2E2 in the hippocampus. Immunofluorescence co-labelling of MeCP2E1 with a neuronal nuclei marker NeuN also showed the majority of MeCP2E1 and MeCP2E2 signals to be localized to NeuN^+^ positive cells in the hippocampus CA1 layer (Figure 4A-A1). Confocal analysis showed similar nuclear patterns for MeCP2E1 and MeCP2E2 in NeuN^+^ nuclei (Figure 4D-D1). We also detected abundant labelling for each cell type-specific marker in the CA1 of female brain (Figure 4B-B1; C-C1). Confocal microscopy imaging analysis was performed and we detected lower levels of both MeCP2E1 and MeCP2E2 signals in GFAP^+^ astrocytes (Figure 4E-E1) and CNPase^+^ oligodendrocytes (Figure 4F-F1) in the hippocampus CA1 layer of adult female brain. As stated earlier, the specificity of the detection of lower levels of MeCP2 isoforms in astrocytes (GFAP^+^), and oligodendrocytes (CNPase^+^) was confirmed by the absence of the specific signals in similar GFAP^+^ and CNPase^+^ cells in the *Mecp2* ^tm1.1Bird y/−^ null mouse brain hippocampus (Figure S4).

**Figure 5:**
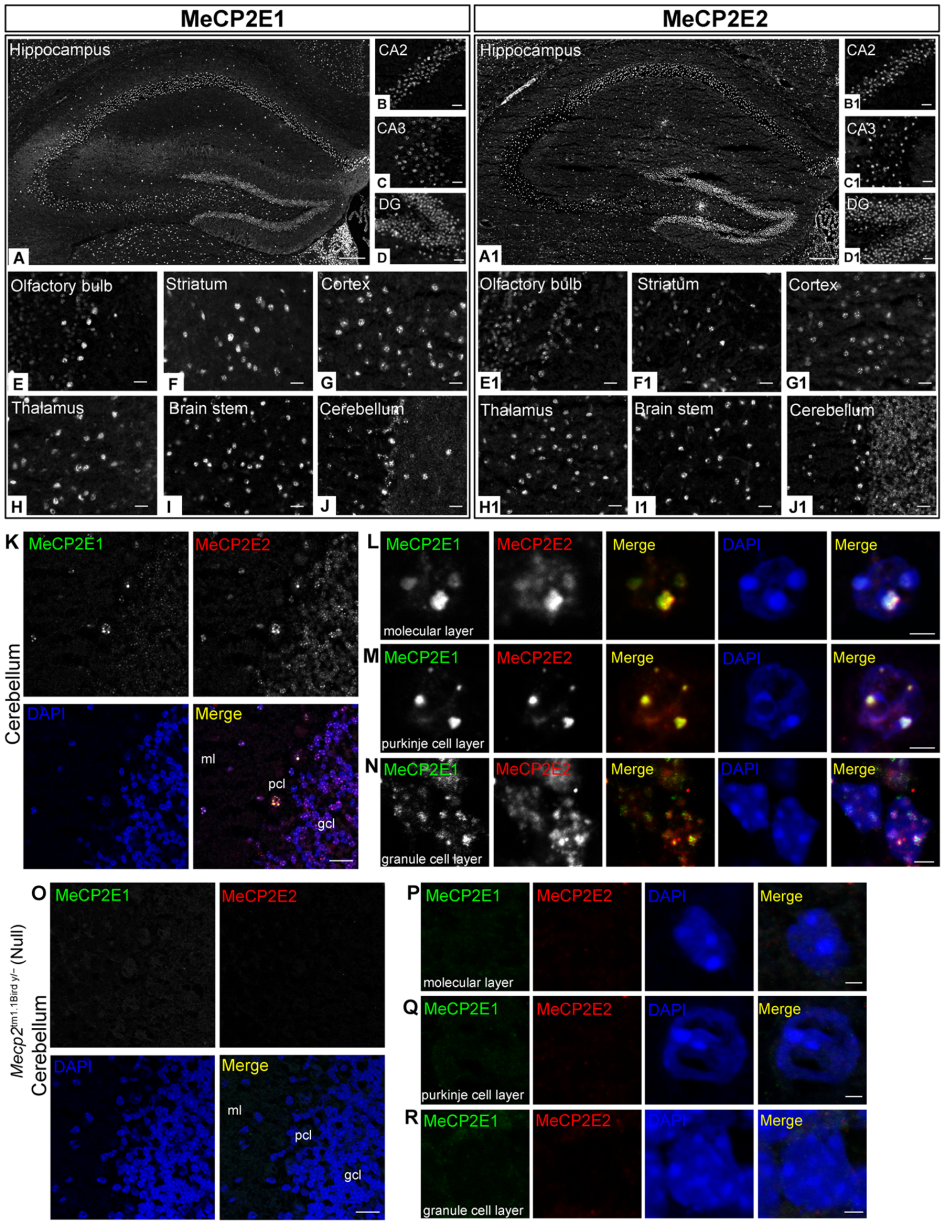
Immunohistochemical detection of MeCP2E1 and MeCP2E2 in the adult mouse brain regions. Left panel **(A**–**J)**: MeCP2E1, and right panel **(A1**–**J1)**: MeCP2E2. Expression of MeCP2 isoforms was analyzed in seven indicated brain regions of the adult mouse brain. **(A**–**A1)** Tiled image of whole hippocampus, and specific regions of hippocampus, **(B**–**B1)** CA2, **(C**–**C1)** CA3, **(D**–**D1)** Dentate gyrus (DG); **(E**–**E1)** Olfactory bulb; **(F**–**F1)** Striatum; **(G**–**G1)** Cortex; **(H**–**H1)** Thalamus; **(I**–**I1)** Brain stem; **(J**–**J1)** Cerebellum. Scale bars represent 200 µm in (A–A1) and 20 µm in (B–J and B1–J1). **(K)** Detection of MeCP2E1 and MeCP2E2 in three layers of the cerebellum; molecular layer (ml), Purkinje cell layer (pcl) and granule cell layer (gcl). Scale bars represent 20 µm. **(L**–**N)** Colocalization of MeCP2E1 and MeCP2E2 in the nuclei in the three layers of the cerebellum; **(L)** molecular layer, **(M)** Purkinje cell layer and **(N)** granular cell layer. Scale bars represent 2 µm. **(O)** Absence of MeCP2E1-, and MeCP2E2-specific signals in three layers of the cerebellum of the *Mecp2* ^tm1.1Bird y/−^ null mouse brain [molecular layer (ml), Purkinje cell layer (pcl) and granule cell layer (gcl)]. Scale bars represent 20 µm. **(P**–**R)** Absence of MeCP2E1/- and MeCP2E2-specific signals in the nuclei in the three layers of the cerebellum of the *Mecp2* ^tm1.1Bird y/−^ null mouse brain; **(P)** molecular layer, **(Q)** Purkinje cell layer and **(R)** granular cell layer. Scale bars represent 2 µm.

We further compared distribution of MeCP2 isoforms in other regions of male hippocampus including CA2, CA3 and dentate gyrus (DG) (Figure 5A-A1). Higher magnification of CA2, CA3 and DG in mouse hippocampus sections showed no obvious differences for MeCP2E1 and MeCP2E2 labelling in these regions (Figure 5B-D; B1-D1) similar to what was observed in the CA1 region. Nuclear labelling was evident in other hippocampus layers surrounding the pyramidal and dentate regions as seen in the low magnification tiled images (Figure 5A-A1).

It has been reported that *Mecp2e1* and *Mecp2e2* transcripts are differentially distributed throughout different mouse brain regions [20]. Additionally, we previously reported the distribution of MeCP2E1 in the adult mouse brain that followed similar pattern to total MeCP2 [21]. However, comparative analysis of MeCP2E1 and MeCP2E2 endogenous protein expression and localization in different brain regions has not been reported to date. To investigate the spatial expression of MeCP2 isoforms, we performed IHC experiments in different regions of the adult mouse brain, namely, the olfactory bulb, striatum, cortex, hippocampus, thalamus, brain stem and cerebellum. Similar to the hippocampus, labelling for both MeCP2 isoforms in other examined brain regions was abundant with no obvious differences in staining patterns in the majority of studied regions (Figure 5E-J, 5E1-J1). In the olfactory bulb the most intense signals detected by anti-MeCP2 isoform-specific antibodies were in the mitral cell layer, presumably in mitral cells (Figure 5E, 5E1). Similar staining intensity was also observed in the nuclei localized within the inner and outer plexiform layers. Lower level intensities were observed for staining of both MeCP2 isoforms in the granule cell layer. Interestingly, while MeCP2E1 and MeCP2E2 labelling was observed in the same juxtaglomerular nuclei of the olfactory bulb, some nuclei were devoid of labelling for both isoforms.

Similar IF labelling patterns of MeCP2 isoforms were observed in dorsal/ventral and medial regions of the striatum (data not shown), as well as in nuclei surrounding fibre-dense regions of caudate putamen in the lateral striatum (Figure 5F, 5F1). Immunofluorescence labelling of MeCP2E1 and MeCP2E2 was detected in all layers of rostral to caudal cerebral cortex as shown here in layers 5–6 (Figure 5G, 5G1). Likewise, positive labelling for both MeCP2 isoforms was observed throughout the thalamus of mouse brain, including medial areas (Figure 5H, 5H1) and the dorsal region underlying the ventral hippocampus (Figure 5A, 5A1), as well as throughout regions of the brain stem, including the medial vestibular nucleus (Figure 5I, 5I1).

Low magnification images of the distribution of MeCP2E1 and MeCP2E2 signals within the cerebellum indicated a higher expression level of MeCP2E2 within the granule cell layer of the cerebellum (Figure 5J and 5J1). To further confirm the differential levels of MeCP2E1 and MeCP2E2 signals, we performed IHC double-labelling for both MeCP2E1 [using a newly developed rabbit polyclonal anti-MeCP2E1 antibody (Figure S5; Note S2)] and anti-MeCP2E2 antibody in sections of male mouse cerebellum. Confocal microscopy, using the same imaging settings, of double immunofluorescence-labelling with rabbit anti-MeCP2E1 and chicken anti-MeCP2E2 antibodies in mouse cerebellum supported our single-labelling data and confirmed differential detection levels of MeCP2E1 and MeCP2E2 in the granule cell layer of mouse cerebellum (Figure 5K). Using confocal microscopy, we show that in the cerebellum sub-regions of molecular layer, Purkinje cell and granule cell layer, MeCP2E1 and MeCP2E2 signals are co-localized with each other at the chromocenters (Figure 5L-N). As expected, no signal was detected for either MeCP2 isoform, when we repeated IHC experiments in the cerebellum of null mouse brain (Figure 5O), indicating the specificity of the detected signals in the WT cerebellum. Accordingly, no signal was detected for either MeCP2 isoform by confocal microscopy of the null mouse brain in any of the three cell layers within the cerebellum (Figure 5P-R).

Taken together, our results demonstrate an overall similar cell type-specific distribution of MeCP2 isoforms between hippocampus region of mouse male and female brain. Although MeCP2E1 and MeCP2E2 signals are mostly identical throughout brain regions, differential abundance of MeCP2E1 and MeCP2E2 exists at least in the granule cell layer of the cerebellum as seen in male brain. Furthermore, both MeCP2 isoforms were detected in all three neural cell types examined in the present study.

### *Mecp2*/MeCP2 isoforms show differential abundance in adult murine brain regions

**Figure 6:**
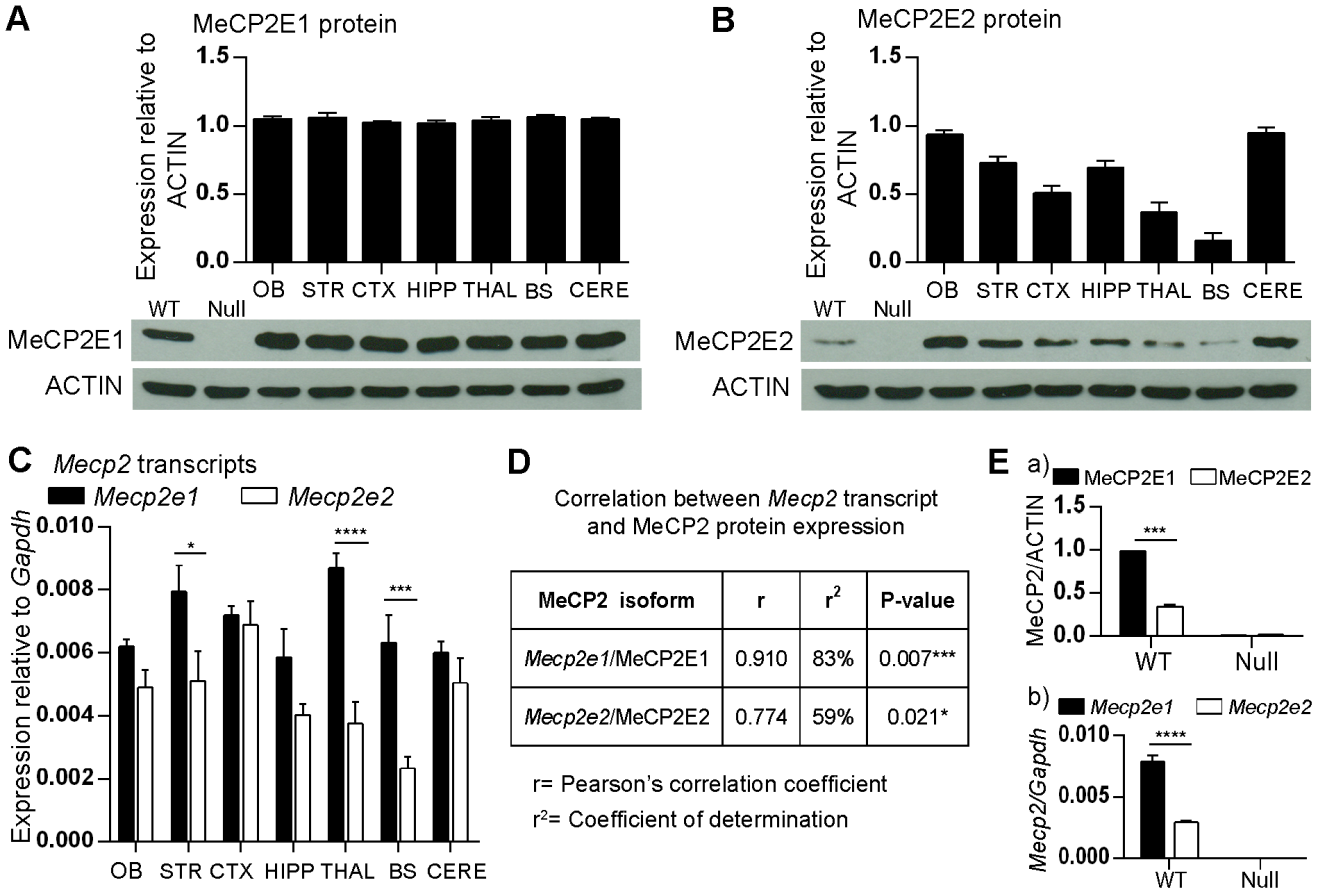
Transcript and protein expression of MeCP2 isoforms in adult mouse brain regions. Expression of *Mecp2*/MeCP2 isoforms was analyzed in the indicated brain regions of the adult male murine brain. **(A)** Western blot (WB) analysis of MeCP2E1 in brain regions. ACTIN was used as a loading control. N = 3±SEM. Whole WT *Mecp2* and null *Mecp2* adult brains were used as controls. **(B)** Similar to A for MeCP2E2. N = 3±SEM. **(C)** Quantitative RT-PCR to detect transcript levels of *Mecp2* isoforms in brain regions. N = 3±SEM. Significant differences between the two isoforms are indicated with P<0.0001****, P<0.001***, P<0.01** or P<0.05*. For A–B, significant differences in the levels of each MeCP2 isoform between different brain regions are found in Table S2. **(D)** Pearson's correlation analysis between *Mecp2* transcript and MeCP2 protein expression. r =  Pearson's correlation coefficient, r^2^ = coefficient of determination, P<0.001*** or P<0.05*. **(E)** (a) Semi-quantitative representation of MeCP2E1 and MeCP2E2 levels in WT *Mecp2* and null *Mecp2* adult brain. N = 3±SEM. (b) Quantitative RT-PCR to detect transcript levels of *Mecp2* isoforms in WT *Mecp2* and null *Mecp2* adult whole brains. N = 3±SEM. Significant differences between the two isoforms are indicated with P<0.0001****, P<0.001***. OB: olfactory bulb, STR: striatum, CTX: cortex, HIPP: hippocampus, THAL: thalamus, BS: brain stem, CERE: cerebellum.

Since we observed highly similar distribution and localization of MeCP2 isoforms by IHC within all the brain regions except for the cerebellum, we next sought to quantify the abundance of MeCP2 isoforms in different brain regions by WB. We used nuclear extracts for these studies, as our WB and immunofluorescence experiments showed the nuclear localization of both MeCP2 isoforms. Expression analysis of MeCP2E1 protein levels showed uniform expression levels across different brain regions that were analyzed (Figure 6A; Table S2). Similar expression profile was seen with *Mecp2e1* transcripts in all the studied brain regions (Figure 6C; Table S2). Pearson's correlation analysis revealed a statistically significant correlation between *Mecp2e1*/MeCP2E1 transcripts and protein levels (r = 0.91, P<0.01) (Figure 6D). In contrast to the results obtained with MeCP2E1, MeCP2E2 showed a differential expression pattern across different brain regions with significantly higher expression in the olfactory bulb and the cerebellum compared to other brain regions (Figure 6B; Table S2). Brain stem showed the lowest expression of MeCP2E2 compared to other examined regions. *Mecp2e2* transcript levels were also differentially expressed in the analyzed brain regions with significant differences between the cortex and thalamus, and cortex and brain stem (Figure 6C; Table S2). Correlation analysis between MeCP2E2 protein and *Mecp2e2* transcript levels revealed a statistically significant correlation between *Mecp2e2*/MeCP2E2 (r = 0.77, P<0.05). As positive and negative controls for the aforementioned analysis, we used whole *Mecp2* WT and null adult brains (adult mice at 6 weeks of age), respectively for analysis of *Mecp2*/MeCP2 isoform-specific expression. As expected, the expression levels of MeCP2E1 were significantly higher (2.8-fold) than that of MeCP2E2 in the WT whole brain, whereas neither isoform was detected in the nuclear extracts of null mouse brain (Figure 6E:a). Similarly, higher *Mecp2e1* transcript levels were detected in the WT brain (2.6-fold), relative to lower *Mecp2e2* levels, while no transcripts were detected in the null brain (Figure 6E:b). These observations further confirm that MeCP2E1 is the major isoform in the adult mouse brain. Detection of total MeCP2 in all these analyzed brain regions were confirmed using a commercial antibody (Figure S3B).

### Differential DNA methylation at the *Mecp2* regulatory elements correlate with the expression of *Mecp2* isoforms in the adult mouse brain regions

**Figure 7:**
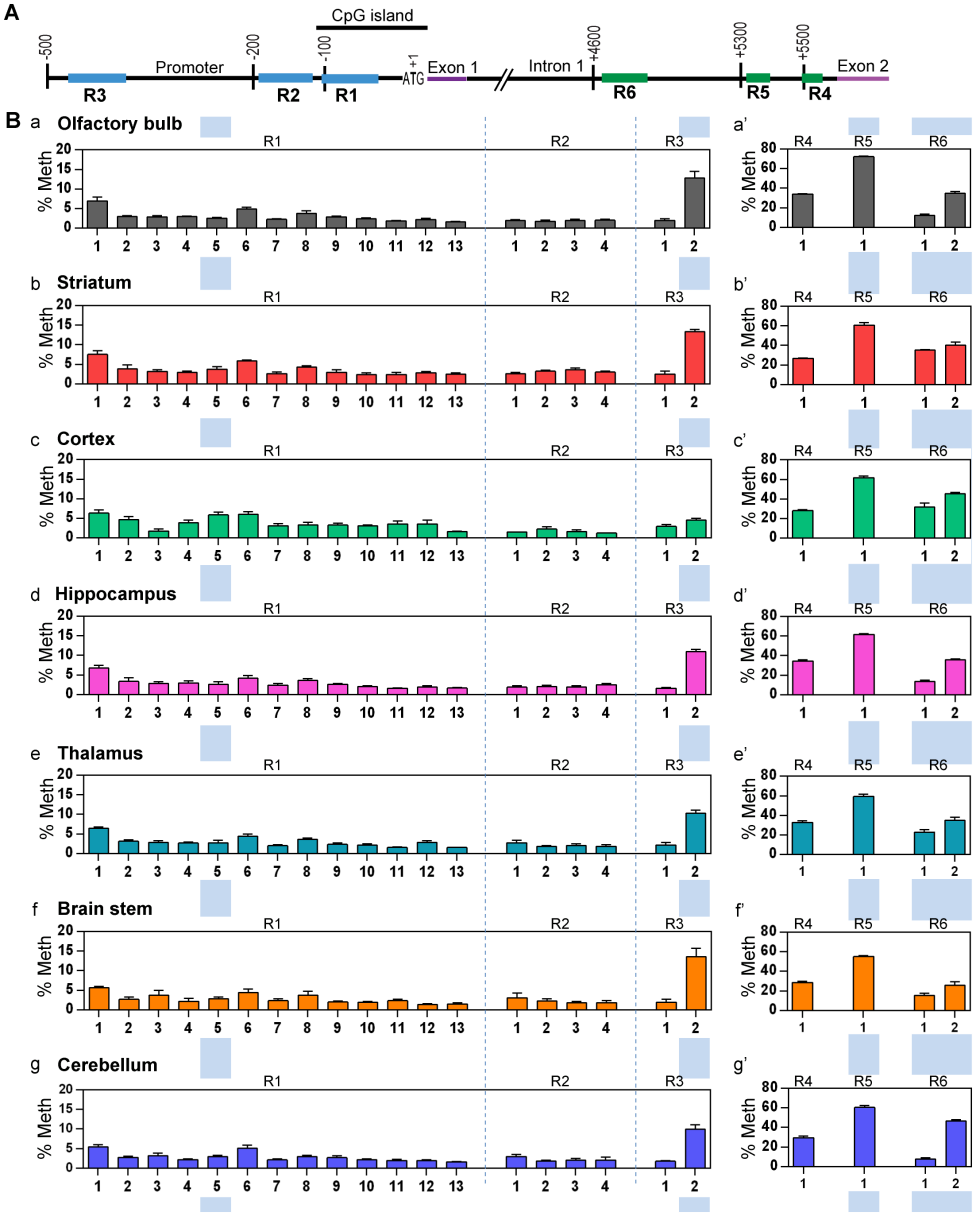
Bisulfite pyrosequencing analysis of DNA methylation status at the *Mecp2* regulatory elements in adult murine brain regions. **(A)** Schematic representation of regions within the *Mecp2* promoter (R1–R3) and intron 1 (R4–R6) (not drawn to scale) [17]. The first ATG of exon 1 is marked +1. **(B)** The graphs represent the percentage methylation (% Meth) observed at the individual CpG sites within analyzed sequences in seven indicated brain regions of adult mouse brain. a-a′: olfactory bulb, b-b′: striatum, c-c′: cortex, d-d′: hippocampus, e-e′: thalamus, f-f′: brain stem and g-g′: cerebellum. N = 5±SEM. Blue shaded regions show statistically significant differences between brain regions. For detailed comparison of statistical analysis, see Table S3.

Previous studies have shown the correlation between the increased *MECP2/Mecp2* promoter methylation and the reduced *MECP2/Mecp2* expression in the brain cortex of human autistic patients [30], and in the brain of stressed mice induced by maternal separation [32]. More recently, we established the correlation of *Mecp2* isoform-specific expression and DNA methylation at the *Mecp2* REs during neural stem cell differentiation and in response to a DNA demethylating drug, Decitabine [17]. These previous reports prompted us to investigate whether there is any correlation between DNA methylation at the *Mecp2* REs and *Mecp2* isoform-specific expression in different brain regions. Therefore, we performed bisulfite pyrosequencing to analyze DNA methylation patterns of three DNA regions within the *Mecp2* promoter (R1-R3: Figure 7A), and three regions within the intron 1 (R4-R6: Figure 7A). These DNA sequences are the same as we previously studied in differentiating neural stem cells from mice [17]. Percentage DNA methylation (% Meth) at individual CpG sites within each region (R1-R6) and the average DNA methylation over the entire DNA regions (R1-R6) were determined.

Between the different brain regions that we analyzed, two CpG sites within the *Mecp2* promoter, namely R1:CpG 5 (∼2.5–3.5%) and R3:CpG 2 (∼2–10%) showed significantly different methylation patterns (Figure 7B; Table S3). No significant changes in DNA methylation patterns were observed at the individual CpG sites within the promoter R2. When the overall/average DNA methylation over the entire R1, R2 and R3 were analyzed, we did not observe any significant difference between the analyzed brain regions (Figure S6; Table S4). Analysis of intron 1 regulatory elements showed significantly different DNA methylation patterns for the analyzed brain regions at three CpG sites. These three CpG sites are as following: R5:CpG 1 (∼10–18%), R6:CpG 1 (∼7–28%), and R6:CpG 2 (∼8–21%) (Figure 7B; Table S3). The percentage DNA methylation at the single CpG site within R4 was consistent in all brain regions (Figure 7B; Table S3). Similar to the individual CpG sites within the intron 1 R6, average methylation over the entire R6 showed significant differences between analyzed brain regions (Figure S6; Table S4).

**Figure 8:**
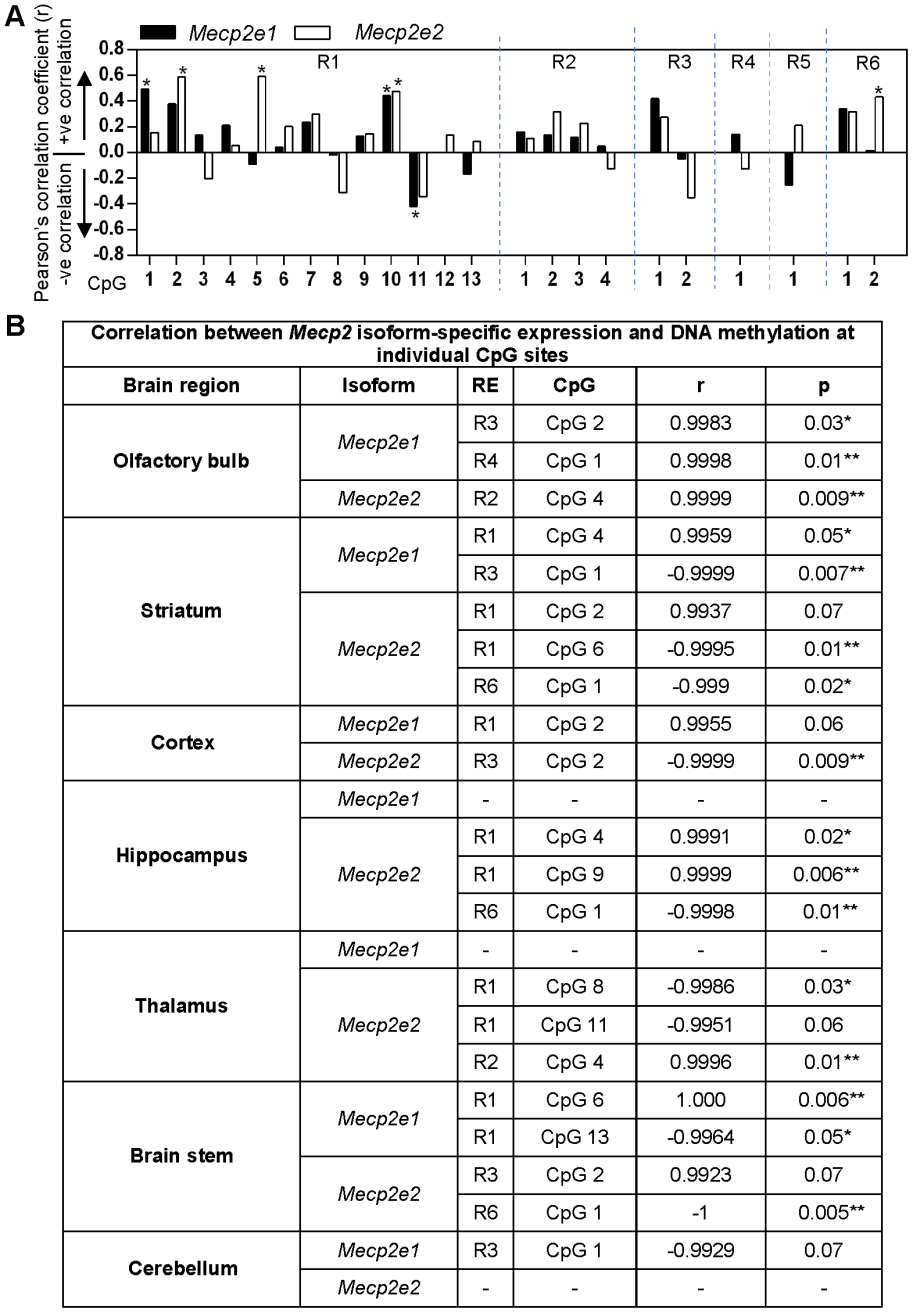
Correlation analysis between DNA methylation at the *Mecp2* regulatory elements and the expression of *Mecp2* isoforms in the adult mouse brain regions. **(A)** Graph represents Pearson's correlation coefficient (r) for correlation between DNA methylation at the *Mecp2* regulatory elements and *Mecp2e1* (black), and *Mecp2e2* (white) expression in seven adult mouse brain regions. Stars (*) indicate statistical significance P<0.05*. N = 3. **(B)** Table representing Pearson's correlation coefficient (r) for correlation between DNA methylation at the *Mecp2* regulatory elements and expression of *Mecp2e1* (black), *and Mecp2e2* (white). Stars (*) indicate statistical significance P<0.05*. N = 3.

In order to investigate the correlation between *Mecp2* isoform-specific expression and differential DNA methylation at the *Mecp2* REs, we performed Pearson's correlation analysis between these variables. We found that specific CpG sites within the studied regions have preferential correlation with individual *Mecp2* isoforms (Figure 8A). For instance, R1:CpG 1 (r = 0.49, P<0.05), R1:CpG 11 (r = 0.42, P≤0.05) and R3:CpG 1 (r = 0.41, P≤0.05) showed significant correlation with *Mecp2e1*, while R1:CpG 2 (r = 0.58, P<0.01), R1:CpG 5 (r = 0.59, P<0.01), R3:CpG 2 (r = 0.35, P = 0.1) and R6:CpG 2 (r = 0.43, P<0.05) showed significant correlation with *Mecp2e2*. Specific CpGs such as R1:CpG 10 showed relatively equal correlation with both *Mecp2* isoforms [*Mecp2e1*: r = 0.44, P<0.05; *Mecp2e2*: r = 0.47, P<0.05].

Since we observed a correlation between DNA methylation and *Mecp2* expression profiles in all brain regions together, we next studied if there was any correlation between DNA methylation and *Mecp2* isoform-specific expression in individual brain regions. Interestingly, in all brain regions *Mecp2e1* and *Mecp2e2* showed differential correlation with specific CpG dinucleotides within the studied regions (Figure 8B). For instance, in the brain regions with significant differences in the expression of *Mecp2* isoforms, such as striatum, thalamus and brain stem, *Mecp2e1* showed preferential correlation with CpGs within R1 and R3 [striatum: R1: CpG 4, r = 0.99, P≤0.05 and R3:CpG 1, r = −0.99, P<0.01; thalamus: no correlation; brain stem: R1:CpG 6, r = 1.0, P<0.01 and R1:CpG 13, r = −0.99, P≤0.05]. On the other hand, *Mecp2e2* showed preferential correlation with CpGs found within R1, R2 and R6 in the same brain regions [striatum: R1:CpG 2, r = 0.99, P<0.05, R1:CpG 6, r = −0.99, P≤0.01 and R6:CpG 1, r = −0.99, P<0.05; thalamus: R1:CpG 8, r = −0.99, P<0.05, R1:CpG 11, r = −0.99, P<0.01 and R2:CpG 1, r = 0.99, P≤0.01; brain stem: R3:CpG 2, r = 0.99, P = 0.07 and R6:CpG 1, r = −1.0, P<0.01].

In summary, these results show that DNA methylation at the *Mecp2* REs correlates with *Mecp2* isoform-specific expression and thus imply a role for DNA methylation in affecting *Mecp2e1* and *Mecp2e2* expression in different regions of the adult mouse brain.

## Discussion

In this study, we report a comparative analysis of *Mecp2*/MeCP2 isoform-specific expression during mouse brain development and in different brain regions of young adult mice at 6 weeks of age. MeCP2 isoforms show significant increase at the protein levels during the early postnatal mouse development (P1–P7). This time period has been reported to coincide with the onset of neuronal maturation and synaptogenesis in several brain regions [33], [34]; thus, the possibility of both MeCP2 isoforms contributing to these processes cannot be ruled out. It is noteworthy that the later onset of MeCP2E2 protein expression (compared to MeCP2E1) might reflect the developmental pattern of a regional, neuronal or cellular subtype in the brain. This is important in light of the knowledge that MeCP2 dysfunction affects different regions of the brain to different extents [38]–[42], suggesting that MeCP2E2 may contribute to normal function of specific types of neurons or other brain cell types. Moreover, the absence of a significant correlation between *Mecp2*/MeCP2 transcript and protein expression of the two isoforms during brain development suggest possible post-transcriptional regulation of *Mecp2* isoforms during development. Differential regulation of *MECP2* expression during human brain development [43], and *Mecp2* isoform-specific expression during mouse brain development has also been suggested before [20].

Recent studies have shown that MeCP2 expression levels are critical to maintain, and higher or lower levels than normal in different brain regions correlate with specific behavioural impairments [44]. Moreover, deletion of *Mecp2* in neurons in specific brain-regions is associated with RTT phenotypes [45]–[50]. This reinforces the requirement for precise levels of MeCP2 expression for normal brain function, as both higher and lower levels of MeCP2 expression (compared to normal) result in neurological dysfunction. Our WB data show that both MeCP2 isoforms are present in the adult mouse brain, with MeCP2E1 showing more uniform expression levels in different brain regions compared to MeCP2E2. Previously, we reported the differential expression of MeCP2E1 in brain regions [21], in which total cell extracts from different brain regions were analyzed. Here, we confirm that similar to MeCP2, MeCP2E1 is also a nuclear protein. Therefore, by using nuclear extracts, we eliminated the influence of cellular size and also nuclear to cytoplasmic ratio, which might not be the same in different cell types, or regions of the brain. Although the significance of the uniform nuclear distribution of MeCP2E1 in brain remains to be elucidated; it might be related to a specialized MeCP2E1 nuclei structural function in different brain regions. Our findings regarding the MeCP2E2 expression patterns suggest that it may contribute to MeCP2 brain region-specific functions, or target genes. This is supported by recent studies where *Mecp2e2* overexpression resulted in different phenotypic consequences in mouse hippocampus and cerebellum [15], [51].

The expression of MeCP2 in neurons, astrocytes and oligodendrocytes has been demonstrated previously [17], [18], [21], [37], [52]. The detection of MeCP2E1 in neurons and astrocytes in the mouse brain is in agreement with our previous report on the detection of MeCP2E1 in embryonic neurons and astrocytes [21]. The contribution of cell type-specific expression of MeCP2 to RTT pathogenesis has been demonstrated previously, in neurons [35], [47], astrocytes [53]–[55], and oligodendrocytes [56], [57]. Therefore, our findings on the expression of MeCP2 isoforms in different cell types of the brain would shed more light on the contribution of MeCP2 isoforms to the pathology of MeCP2-related neurodevelopmental disorders including Rett Syndrome.

The nuclear architecture, number, size and distribution of chromocenters have been shown to be dependent on the cell type, the state of differentiation and stage of cell cycle [58], [59]. Given that MeCP2 is a major contributor in chromatin architecture and chromocenters clustering [8], it is possible that its localization to chromocenters is different depending on the aforementioned factors. However, this has not been studied before. Our observations on the differences in the localization of MeCP2 isoforms to chromocenters between neurons and glial cells provide insights on the potential differences in cell type-specific MeCP2 localization and would require further investigations.

Interestingly, semi-quantitative WB analysis showed similar protein expression levels of MeCP2 isoforms in the adult mouse cerebellum, but further IHC characterization revealed differential levels of MeCP2 isoform-specific detection in sub-regions of the cerebellum. Our results indicated that MeCP2E2 is slightly more abundant in the granule cell layer of the cerebellum compared to MeCP2E1, and supports a recent study demonstrating increased neuronal apoptosis of cerebellar granule neurons due to elevated levels of *Mecp2e2* but not *Mecp2e1* [15]. Thus, our findings confirm that MeCP2 isoforms are also differentially localized in this part of the brain at the protein levels. This is also important with regard to disease pathophysiology, as duplication of the *MECP2* gene has been found to cause cerebellar degeneration in humans [60]. However, it remains unknown if the atrophy is specific to particular neuronal-subtypes in the cerebellum. Therefore, it is possible that MeCP2E2 contributes to this phenotype that is observed in MeCP2 duplication syndrome. Dysfunction of MeCP2 in the cerebellum has been shown to cause altered expression many of genes [61], [62]. The detected differentiation distribution of MeCP2 isoforms in the cerebellum might be helpful to understand the contribution of individual MeCP2 isoforms in cerebellar functions and gene expression.

As mentioned earlier, in mice, as well as in humans, reduced *Mecp2/MECP2* expression in the brain cortex is correlated with increased DNA methylation at the *Mecp2* promoter. We observed dynamic correlation between *Mecp2* isoform-specific expression and DNA methylation at specific *Mecp2* REs in the neural stem cells isolated from embryonic forebrain [17]. Here for the first time, our study reports the correlation between DNA methylation at the *Mecp2* REs (promoter and intron 1) and the endogenous expression pattern of *Mecp2* isoforms, in seven different brain regions in the adult mice. Interestingly, DNA methylation at most CpG sites within the studied REs remained consistent, with the exceptions of R3:CpG 2, R5:CpG 1, R6:CpG 1, and R6:CpG 2, which showed significant differences in DNA methylation between the analyzed brain regions. The differences in DNA methylation at particular CpGs within specific REs suggest the potential functional significance of these CpGs in regulating *Mecp2* isoform-specific expression in certain brain regions. However, the functional significance of DNA methylation at these CpG sites in regulating *Mecp2* isoforms remains to be determined. Moreover, the bisulfite pyrosequencing analysis does not differentiate between 5 mC and 5 hmC methyl marks [63], [64], which generally have opposing functions on gene expression. The observation of positive correlations between *Mecp2* expression and DNA methylation at specific CpG sites (Figure 8) as opposed to the general negative/inverse correlation between DNA methylation (5 mC) and gene expression also suggest the possibility of differential 5 mC and 5 hmC enrichment at these CpG sites. Utility of techniques that differentiate between 5 mC and 5 hmC methylation patters such as methylated or hydroxymethylated DNA immunoprecipitation (MeDIP/hMeDIP) would be helpful in determining the precise functions of the differences in methylation patters at the *Mecp2* REs we observed.

Another interesting observation was that DNA methylation at the *Mecp2* promoter regions ranged between 2–15%, whereas that of intron 1 regions was between 25–75%. The differential DNA methylation that was observed between the *Mecp2* promoter and intron 1 elements might imply that different levels of regulation are exerted for proper expression of *Mecp2* isoforms in the brain. The intron 1 sequences analyzed in our study overlaps with a previously reported silencer element. The same silencer element is suggested to be potentially important in alternative splicing or tissue-specific expression of MeCP2 [27]. We observed significant positive correlation between R4 DNA methylation and *Mecp2e1* expression in the olfactory bulb, while other brain regions such as striatum, hippocampus and brain stem showed a significant negative correlation between R6 DNA methylation and *Mecp2e2* expression, thereby providing insights on the potential differential impact of these intron 1 elements on *Mecp2* isoforms. The observed correlations between DNA methylation at the *Mecp2* promoter regions (R1–R3) and the expression of both *Mecp2* isoforms, suggest that the *Mecp2* promoter may influence both *Mecp2e1* and *Mecp2e2*. These indicate that DNA methylation may impact individual *Mecp2* isoforms differently, depending on the brain region.

The promoter regions studied here harbour several putative binding sites for regulatory proteins such as Sp1 and CTCF [29], which have been previously shown to be involved in regulating *MECP2/Mecp2* expression [31], [65]._ENREF_27 It is also known that the binding of CTCF and Sp1 are dependent on the DNA methylation status at their corresponding binding sites [66]–[69]. Therefore, it is possible that DNA methylation at the studied sequences might be important for binding of regulatory proteins such as CTCF and Sp1. Taken together, our findings provide fundamental insight towards a better understanding of the tight regulation of *Mecp2* isoforms in the brain.

## Materials and Methods

### Ethics statement

Experiments were conducted in accordance with the standards of the Canadian Council on Animal Care with the approval of the Office of Research Ethics at the University of Manitoba. All experiments were conducted in accordance with animal experimentation guidelines (University of Manitoba). *Mecp2* knockout/null mice (B6.129P2(C)-*Mecp2^tm1.1Bird^*/J) (hereafter referred to as *Mecp2* ^tm1.1Bird y/−^ for the null mice) were purchased from the Jackson Laboratory (USA) along with their wild type (WT) counterparts. All experimental procedures outlined here were reviewed and approved (protocol number 12-031/1) by the University of Manitoba Bannatyne Campus Protocol Management and Review Committee.

### Generation of chicken polyclonal MeCP2E2 and rabbit polyclonal MeCP2E1 isoform-specific antibodies

Conserved sequences between human and mouse MeCP2 protein, in the N-termini of MeCP2E2 (VAGMLGLREEKS) was selected as peptide antigen for polyclonal antibody production in chicken. The peptide sequence for developing anti-MeCP2E1 antibody is the same as previously reported [21]. The anti-MeCP2 isoform-specific immunoglobulins were isolated by peptide affinity purification.

### Quantitative Real Time PCR (qRT-PCR)

Total RNA from brain regions and brains at developmental stages were extracted using RNAeasy Mini Kit (Qiagen Canada Inc., 74134) and converted to cDNA using Superscript III Reverse Transcriptase (Life Technologies Inc., 18080-044), as reported previously [17], [70]–[73]. Quantitative RT-PCR was carried out as described previously using SYBR Green-based RT^2^ qPCR Master Mix (Applied Biosystems, 4367659) in a Fast 7500 Real-Time PCR machine (Applied Biosystems) [17], [18], [70]. Transcript levels of *Mecp2e1* and *Mecp2e2* were examined using gene specific primers (Table S5). PCR program for *Mecp2* consisted of initial denaturation at 95°C for 3 minutes (min) followed by 35 cycles of 1 min at 95°C, 30 seconds (s) at 60°C, and 45 s at 72°C, and a final extension step at 72°C for 10 min. The threshold cycle values (Ct) for each gene was normalized against the housekeeping gene *Gapdh* to obtain ΔCt values for each sample. Relative quantification of gene expression was carried out by calculating 2^−ΔCt^ of each sample. Analysis was performed using Microsoft Excel 2010 and GraphPad Prism 6.0. Two-Way ANOVA was used to calculate significant differences between different brain regions.

### Immunofluorescence and immunohistochemistry

Immunofluorescence antibody detection for cultured NIH3T3 cells was carried out as described earlier [17], [21], [70], using antibodies in Table S6 and S7. Briefly, cultured NIH3T3 cells (ATCC, CRL-1658) on coverslips were washed with phosphate buffered saline (PBS, GIBCO, 14190-136) and fixed in 4% formaldehyde. Fixed cells were then permeabilized with 2% NP40 in PBS for 10 min, followed by preblocking with 10% normal goat serum (NGS, Jackson Immunoresearch Laboratories Inc., 005-000-121) in PBS for 1 h. Primary antibodies were diluted in PBS with 10% NGS and the cells were incubated in primary antibodies overnight at 4°C followed by three washes with PBS. Secondary antibodies diluted in 10% NGS were added to the cells for 1 h, followed by three washes with PBS. Coverslips were mounted on glass slides with Prolong Gold antifade (Molecular Probes, P36930) containing 2 µg/ml 4′,6-diamidino-2-phenylindole (DAPI) (Calbiochem, EMD Millipore, 268298) counter-stain.

Immunohistochemistry (IHC) experiments for adult murine brain were done as described earlier [21]. Briefly, brain tissues were fixed in ice-cold freshly de-polymerized paraformaldehyde (0.16 M sodium phosphate buffer, pH 7.4 with PFA). Subsequently, tissue blocks were incubated in cryoprotectant (25 mM sodium phosphate buffer, pH 7.4, 10% sucrose, 0.04% NaN3) at 4°C for approximately 24 h. Cryosections were processed on to gelatinized slides and stored at −20°C. Prior to IHC experiments, tissues were permeabilized with 0.3% Triton X-100 Tris-buffered saline (TBS-Tr) (50 mM Tris-HCl, pH 7.6, containing 1.5% NaCl) solution. The slides were then pre-blocked with NGS in TBS-Tr and incubated with appropriate primary antibodies diluted in TBS-Tr/serum overnight at 4°C. Secondary antibodies were diluted in TBS-Tr/serum and applied, followed by washes using TBS-Tr and Tris-HCl buffer (50 mM, pH 7.4). Coverslips were prepared after incubation with 0.2 µg/ml DAPI counterstain, washes with Tris-HCl, and application of Prolong Gold (Life Technologies Inc., P36930) antifade.

IF signals were detected using an Axio Observer Z1 inverted microscope and LSM710 confocal microscope (Carl Zeiss Canada Ltd.), as previously described [17], [21], [70]. Images were obtained using Zen Blue 2011, 2012 and Zen Black 2011 (Carl Zeiss Canada Ltd.) software and assembled using Adobe Photoshop C5 and Adobe Illustrator C5.

### Western blot

Nuclear extraction from cultured cells, brain regions, brains from different developmental stages were carried out using the NE-PER Nuclear and Cytoplasmic Extraction Kit (Thermo Scientific Inc., 78835) as previously reported [17], and per manufacturer's instructions. Western blot experiments were conducted according to previously described protocols [17], [74]–[76], and quantification of the signals was performed as previously reported [17], [21], [70]. ACTIN or GAPDH was used as a loading control. Primary and secondary antibodies used for WB are listed in the Table S6 and S7, respectively. For detection of both MeCP2E1 and MeP2E2, similar amount of nuclear protein extracts were loaded. However, different exposure times for anti-MeCP2E1 and E2 antibodies were used, as the efficiency of these antibodies were different.

### Bisulfite pyrosequencing and correlation analysis

DNA samples were isolated from five independent animals (N = 5) using the DNeasy Blood and Tissue kit (Qiagen, 69506), as reported [17], and per manufacturer's instructions. Primer design and DNA bisulfite pyrosequencing analysis was performed as a paid service by SickKids Hospital, Toronto, Canada, as described elsewhere [17]. For DNA methylation analysis, three regions in the *Mecp2* promoter and three regions in the *Mecp2* intron 1 were selected (Figure 7A) based on our previous report [17]. The primers used for Bisulfite pyrosequencing are listed in Table S8. The percentage methylation at individual CpG sites as well as the average methylation over entire regions was determined. The analysis and representation of DNA methylation profiles were performed according to our previous report [17]. Briefly, the statistical differences in DNA methylation patterns between the 7 brain regions were determined by Two-way ANOVA. Differences with P-values lower than 0.05*, 0.01**, 0.001*** and 0.0001**** were considered statistically significant. In order to determine the relationship between DNA methylation and *Mecp2* expression, correlation analysis between DNA methylation at the *Mecp2* regulatory elements and expression of *Mecp2* isoforms was performed using the Pearson's correlation analysis and linear regression as described in our previous study [17]. The correlation coefficients (r) were calculated between DNA methylation at individual CpG sites and all brain regions together, or individual brain region separately. The strength of correlation was considered as following; weak 0<r<0.3, moderate 0.3<r<0.4, strong 0.4<r<0.7, very strong 0.7<r<1.0. The negative “r” value indicates an inverse/negative correlation where as positive “r” value indicates direct/positive correlation. Statistical significance was determined at P<0.05.

### Statistical Analysis

Graphical representations and statistical analysis were performed using GraphPad Prism software (GraphPad Software, Inc.). All graphs represent the average from three independent experiments (N = 3) for gene and protein expression analysis and five independent experiments (N = 5) for DNA methylation analysis. The error bars indicate standard error of mean (SEM). One way ANOVA was performed to evaluate statistically significant mean differences among the brain regions and different-aged mice for individual MeCP2 protein isoforms and mRNA transcripts separately. Two-way ANOVA was performed to evaluate statistically significant mean differences between MeCP2E1 and MeCP2E2 protein and mRNA transcripts (for each region and time point analyzed). For DNA methylation analysis between different brain regions, Two-way ANOVA was performed. For each analysis, we determined the significance levels at P<0.05*, P<0.01**, P<0.001***, and P<0.0001****. Pearson's bivariate and linear regression analyses were performed to evaluate the correlation between MeCP2 protein and transcript levels, as well as DNA methylation at the individual CpG sites and expression of *Mecp2* isoforms.

## Conclusion

In this study, we present a comparative analysis of MeCP2 isoforms during mouse brain development and in the adult mouse brain. We show that MeCP2E1 and MeCP2E2 are expressed in neurons, astrocytes and oligodendrocytes in the brain hippocampus of both male and female brains. Our results also indicate that MeCP2 isoforms display considerable variations with respect to temporal expression during mouse brain development, as well as brain region-specific expression in the adult mice. Furthermore, we report the correlation between the expression of *Mecp2* isoforms and DNA methylation at specific regulatory elements within the *Mecp2* promoter and intron 1 in adult mouse brain regions. We conclude that MeCP2 isoforms are differentially expressed regionally and temporally within the brain, with the possible involvement of DNA methylation at regulatory elements found within the *Mecp2* promoter and intron 1.

## Supporting Information

Figure S1 MeCP2E1 and MeCP2E2 are present in the brain nuclear extracts. **(A)** Detection of MeCP2E1 in the nuclear, but not cytoplasmic extracts from adult mouse brain. Increasing amounts of nuclear and cytoplasmic protein extracts were used. **(B)** Same as A, for MeCP2E2. Membranes are re-probed with GAPDH as a loading control.
(TIF)

Figure S2 Additional controls for anti-MeCP2E2 antibody validation. **(A)** Western blot experiments with Phoenix cell extracts from non-transfected cells (NT), and *MECP2E2* transfected cells (E2-T), probed with the anti-MeCP2E2 antibody after pre-incubation with increasing concentrations of peptide (0%, 0.1%, 1%, and 5%, of peptide as compared to the amount of antibody used). **(B)** Negative controls for immunofluorescence detection of; a) MeCP2E2 and C-MYC in non-transduced NIH3T3 cells, and b) absence of signals in primary omission controls with Rhodamine Red X (RRDX) and FITC in *MECP2E2* transduced NIH3T3 cells. Scale bars represent 10 µm.
(TIF)

Figure S3 Detection of total MeCP2 in mouse brain. **(A)** Detection of total MeCP2 during mouse brain development. **(B)** Detection of total MeCP2 in adult mouse brain regions. ACTIN was used as a loading control. N = 3. OB: olfactory bulb, STR: striatum, CTX: cortex, HIPP: hippocampus, THAL: thalamus, BS: brain stem, CERE: cerebellum.
(TIF)

Figure S4 Absence of MeCP2E1- and MeCP2E2-specific signals in the GFAP^+^ and CNPase^+^ cells of the *Mecp2* ^tm1.1Bird y/−^ null mouse brain hippocampus. Left panel **(A-B)**: MeCP2E1 and, Right panel **(A1-B1)**: MeCP2E2. Absence of the detection of MeCP2 isoforms in **(A-A1)** astrocytes (GFAP^+^), and **(B-B1)** oligodendrocytes (CNPase^+^). Scale bars represent 2 µm. All are confocal images of single nuclei.
(TIF)

Figure S5 Validation of the custom-made rabbit MeCP2E1 antibody. **(A)** Western blot experiment to detect MeCP2E1 expression in control non-transfected (NT), *MECP2E1* transfected (E1-T), *MECP2E2* transfected (E2-T), and *MECP2E1* pre-incubated with the antigenic peptide. Anti-MYC labelling was used as a positive control. GAPDH labelling was used as a loading control. **(B)** Detection of MeCP2E1 by immunofluorescence in NIH3T3 cells transduced with a) *MECP2E1* or b) *MECP2E2*. Scale bars represent 10 µm.
(TIF)

Figure S6 Bisulfite pyrosequencing analysis of average DNA methylation at the *Mecp2* regulatory elements in adult murine brain regions. The graph represents the average percentage methylation (% Meth) observed over the entire regions in seven brain regions of the adult mouse brain. N = 5±SEM. For detailed comparison of statistical analysis, see Table S4.
(TIF)

Note S1 Generation and validation of chicken polyclonal MeCP2E2 antibody.
(DOCX)

Note S2 Generation and validation of rabbit polyclonal MeCP2E1 antibody.
(DOCX)

Table S1 Differences of the expression of *Mecp2*/MeCP2 isoforms across developmental stages.
(DOCX)

Table S2 Differences of the expression of *Mecp2*/MeCP2 isoforms between brain regions.
(DOCX)

Table S3 Comparison of percentage methylation differences at the individual CpG sites between brain regions.
(DOCX)

Table S4 Comparison of average percentage methylation differences between brain regions.
(DOCX)

Table S5 List of primers used in qRT-PCR.
(DOCX)

Table S6 Primary Antibodies.
(DOCX)

Table S7 Secondary antibodies.
(DOCX)

Table S8 List of primers used in bisulfite pyrosequencin.
(DOCX)
