# Correction: Brain Region-Specific Expression of MeCP2 Isoforms Correlates with DNA Methylation within *Mecp2* Regulatory Elements

**DOI:** 10.1371/journal.pone.0101030

**Published:** 2014-07-01

**Authors:** The PLOS ONE Staff

The Figure panel 2C (loading control), Supplementary Figure panels S1A (loading control) and S3A (upper panel) reported in Olson *et al*., 2014 [1] are incorrect. The GADPH loading control in Figure 2C is the mirror image of the ACTIN loading control in Figure 1C of a previous publication [2], and the upper panel of Figure S3A is the same as the upper panel of Figure 1A in the current paper. The GAPDH loading control in Figure S1A is a processed image (2° counter clockwise rotation) from the loading control of a Western blot, from which lanes 3 and 4 correspond to the loading control in Figure 2G in the current paper.

**Figure 2:**
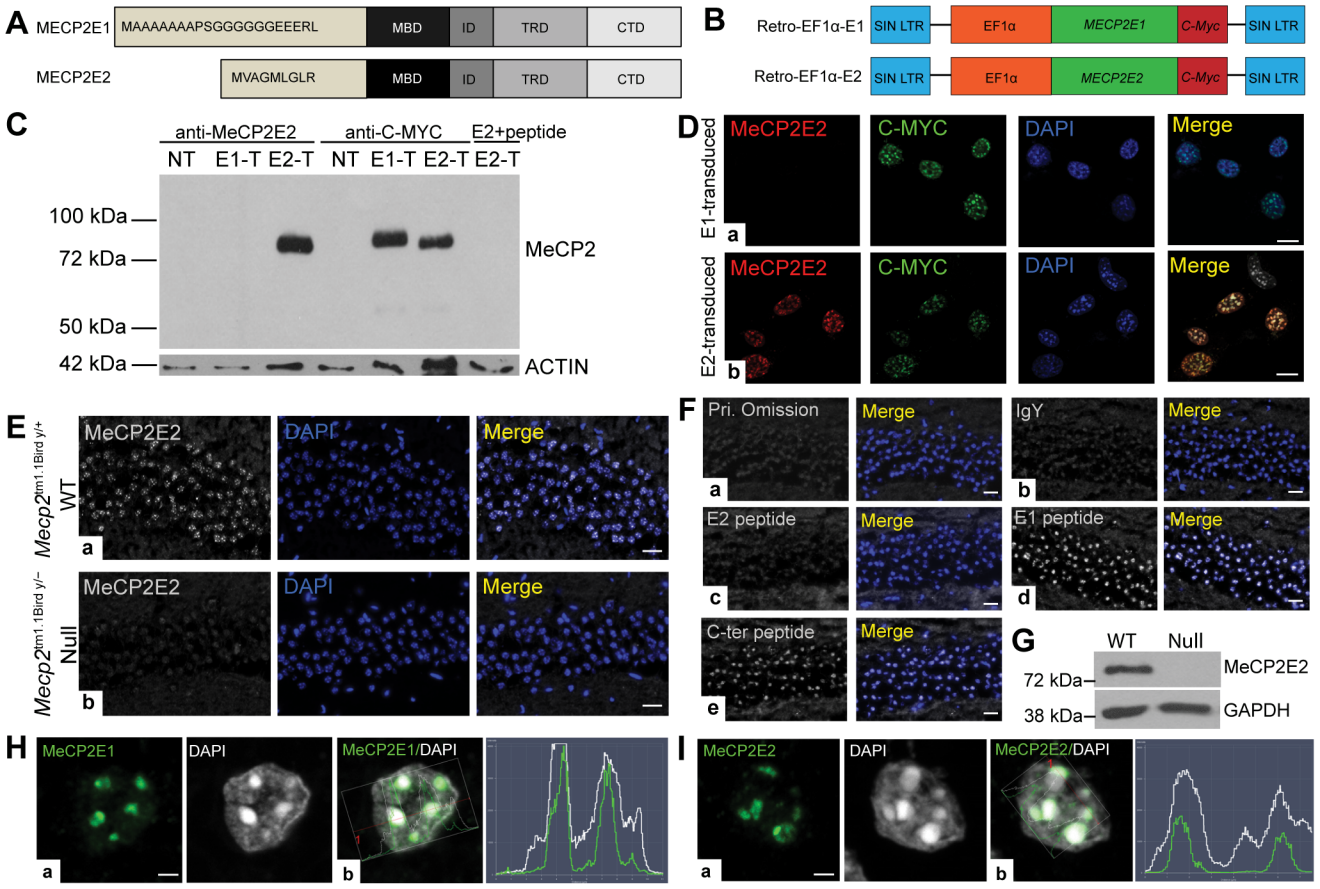
Validation of anti-MeCP2E2 antibody and detection of MeCP2 isoforms in mouse brain. (**A**) Schematic representation of MeCP2E1 and MeCP2E2 protein structures, differing only in their N-terminal sequences. MBD: methyl binding domain, ID: intervening domain, TRD: transcriptional repression domain, CTD: C-terminal domain (adapted from [21]). (**B**) Schematic*s* of *MECP2E1* (Retro-EF1α-E1) and *MECP2E2* (Retro-EF1α-E2) retroviral vectors with C-MYC tag that were used for transfection of Phoenix cells (in C) and transduction of NIH3T3 cells (in D) (adapted from [18]). (**C**) Western blot (WB) experiment to detect MeCP2E2 expression in control non-transfected (NT), *MECP2E1* transfected (E1-T), *MECP2E2* transfected (E2-T), and E2-T pre-incubated with E2 antigenic peptide. Anti-MYC labelling was used as a positive control. (**D**) MeCP2E2 detection by immunofluorescence staining in transduced NIH3T3 cells with either a) *MECP2E1*, or b) *MECP2E2* retroviral vectors. **(E)** Detection of endogenous MeCP2E2 by immunohistochemistry in the CA1 region of adult mouse hippocampus from a) wild type (WT) (*Mecp2* ^tm1.1Bird y/+^), and b) null (*Mecp2* ^tm1.1Bird y/-^) *Mecp2* mice. **(F)** Controls to verify the specificity of anti-MeCP2E2 by IHC in the adult mouse brain; a) primary omission, b) anti-MeCP2E2 incubation with IgY, pre-incubation of the newly generated anti-MeCP2E2 antibody with the antigenic peptide against c) MeCP2E2, d) MeCP2E1, e) C-terminus of MeCP2. **(G)** Western blot to detect MeCP2E2 in the WT adult mouse brain and *Mecp2* null mice. GAPDH was used as a loading control. **(H)** a) Confocal images of MeCP2E1 in WT adult mouse brain hippocampus CA1 region. b) Signal intensity profile analysis indicates the enrichment of MeCP2E1 at the DAPI-rich heterochromatin regions of nuclei. **(I)** a) Confocal images of MeCP2E2 in WT adult mouse brain hippocampus CA1 region. b) Signal intensity profile analysis of MeCP2E1 and DAPI co-localization indicating MeCP2E2 detection at the DAPI-rich heterochromatin regions of nuclei. Scale bars represent 20 µm in D-E, 10 µm in D, and 2 µm in H-I.

The authors apologize for these errors, which happened during the assembly of the figures. Please refer to the corrected Figure 2 here, and the corrected Figures S1 and S3 in the Supporting Information section of this correction. The raw data corresponding to each corrected figure panel is also included. These corrections do not affect the results or conclusions reported in the article.

The authors also correct the following statement in the Introduction:

**Paragraph 2 on page 2:** “Due to the lack of anti-MeCP2E2 antibodies, comparative analysis of both MeCP2 isoforms at the protein levels in any system has not been reported to date.”

While this manuscript was under review, an article was published by Yasui *et al*. in *Human Molecular Genetics*, which did report MeCP2E1 and E2 at the protein level in mouse brain [3]. This study was cited as reference 22 but the statement in the Introduction should have been revised to reflect the findings by Yasui *et al*. In addition, a publication by Kaddoum *et al*. reported an analysis of expression of the MeCP2 isoforms in mouse brain [4].

The authors would like to revise the statement in the Introduction to read as below:

“Dr. Dag Yasui’s group, in collaboration with our lab, has recently reported the expression of MeCP2E1 and MeCP2E2 in the mouse brain [22]. Kaddoum *et al*. also reported an expression analysis of the MeCP2 isoforms in mouse brain. However, a comparative transcript and protein correlation analysis of the two *Mecp2*/MeCP2 isoforms in different brain regions and during mouse brain development has not been reported previously.”

## Supporting Information

File S1. Raw Data for Figure 2C

Figure S1. Supplementary Figure S1 with the corrected panel S1A

File S2. Raw Data for Supplementary Figure S1A

Figure S3. Supplementary Figure S3 with the corrected panel S3A

File S3. Raw Data for Supplementary Figure S3A
