# Supplementary figures and images for: Brain Region-Specific Expression of MeCP2 Isoforms Correlates with DNA Methylation within Mecp2 Regulatory Elements
Source: PLoS One. 2014 Mar 3;9(3):e90645. doi: 10.1371/journal.pone.0090645 (PMC3940938; doi:10.1371/journal.pone.0090645)

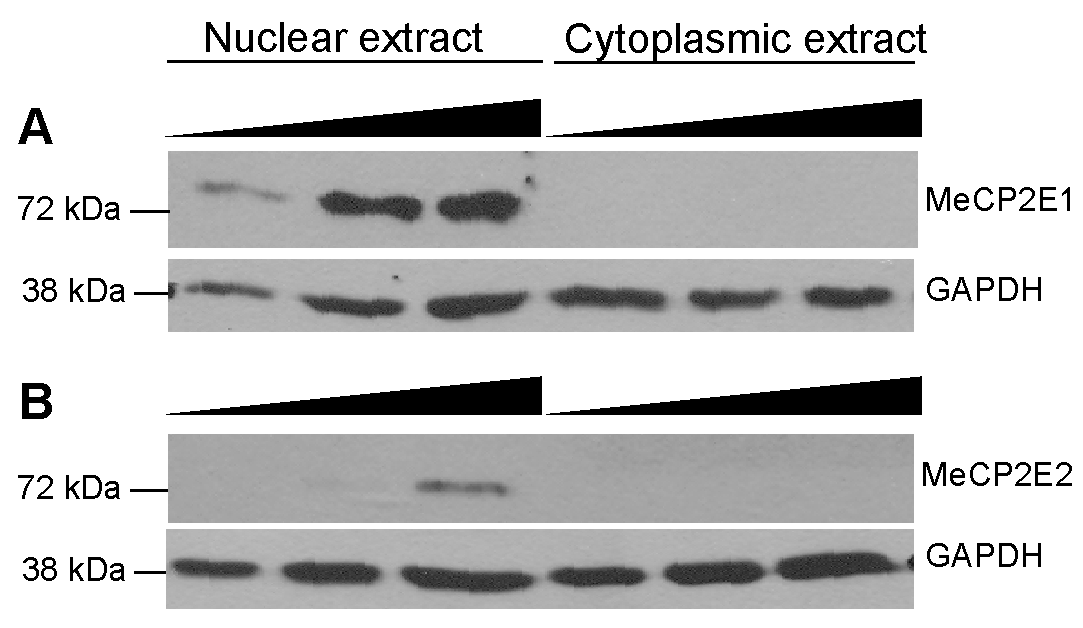

Supplement: Figure S1 — MeCP2E1 and MeCP2E2 are present in the brain nuclear extracts. (A) Detection of MeCP2E1 in the nuclear, but not cytoplasmic extracts from adult mouse brain. Increasing amounts of nuclear and cytoplasmic protein extracts were used. (B) Same as A, for MeCP2E2. Membranes are re-probed with GAPDH as a loading control. (TIF) [file pone.0090645.s001.tif]

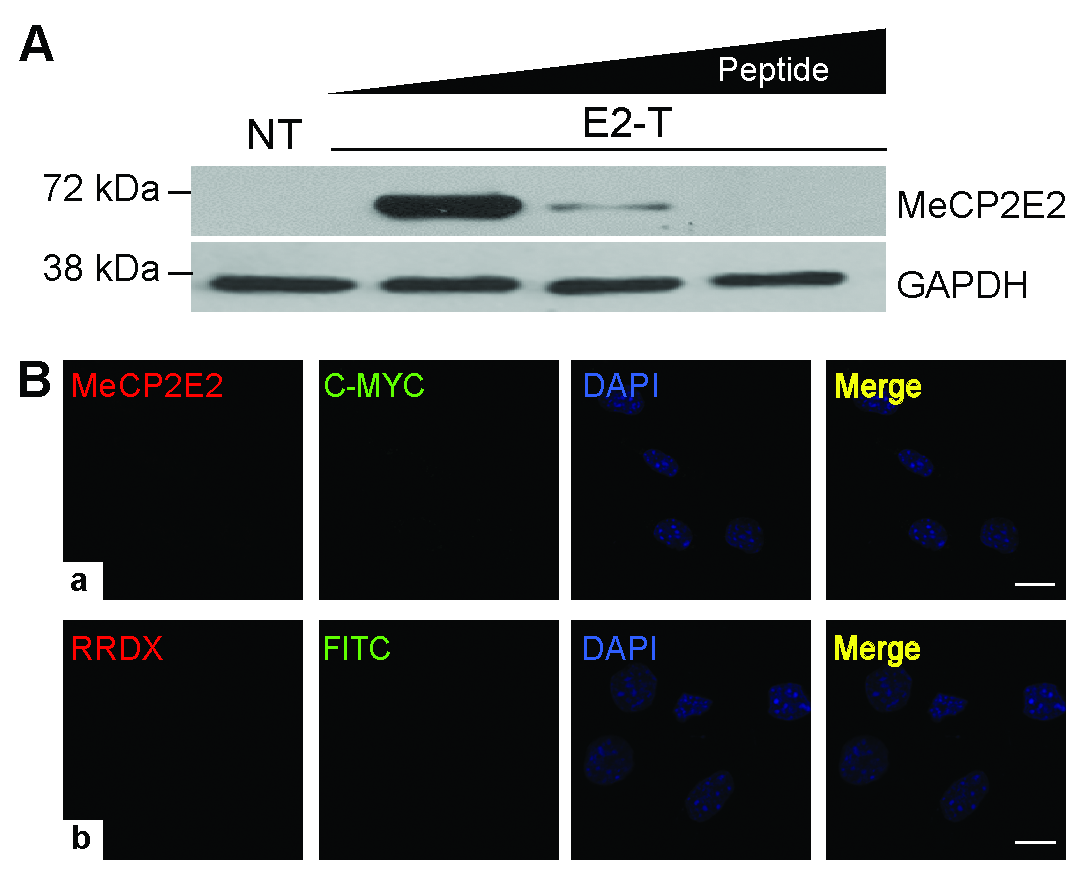

Supplement: Figure S2 — Additional controls for anti-MeCP2E2 antibody validation. (A) Western blot experiments with Phoenix cell extracts from non-transfected cells (NT), and MECP2E2 transfected cells (E2-T), probed with the anti-MeCP2E2 antibody after pre-incubation with increasing concentrations of peptide (0%, 0.1%, 1%, and 5%, of peptide as compared to the amount of antibody used). (B) Negative controls for immunofluorescence detection of; a) MeCP2E2 and C-MYC in non-transduced NIH3T3 cells, and b) absence of signals in primary omission controls with Rhodamine Red X (RRDX) and FITC in MECP2E2 transduced NIH3T3 cells. Scale bars represent 10 µm. (TIF) [file pone.0090645.s002.tif]

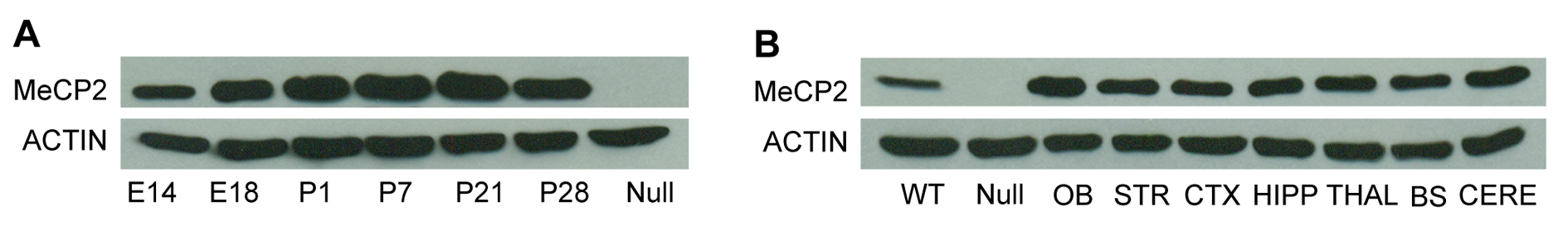

Supplement: Figure S3 — Detection of total MeCP2 in mouse brain. (A) Detection of total MeCP2 during mouse brain development. (B) Detection of total MeCP2 in adult mouse brain regions. ACTIN was used as a loading control. N = 3. OB: olfactory bulb, STR: striatum, CTX: cortex, HIPP: hippocampus, THAL: thalamus, BS: brain stem, CERE: cerebellum. (TIF) [file pone.0090645.s003.tif]

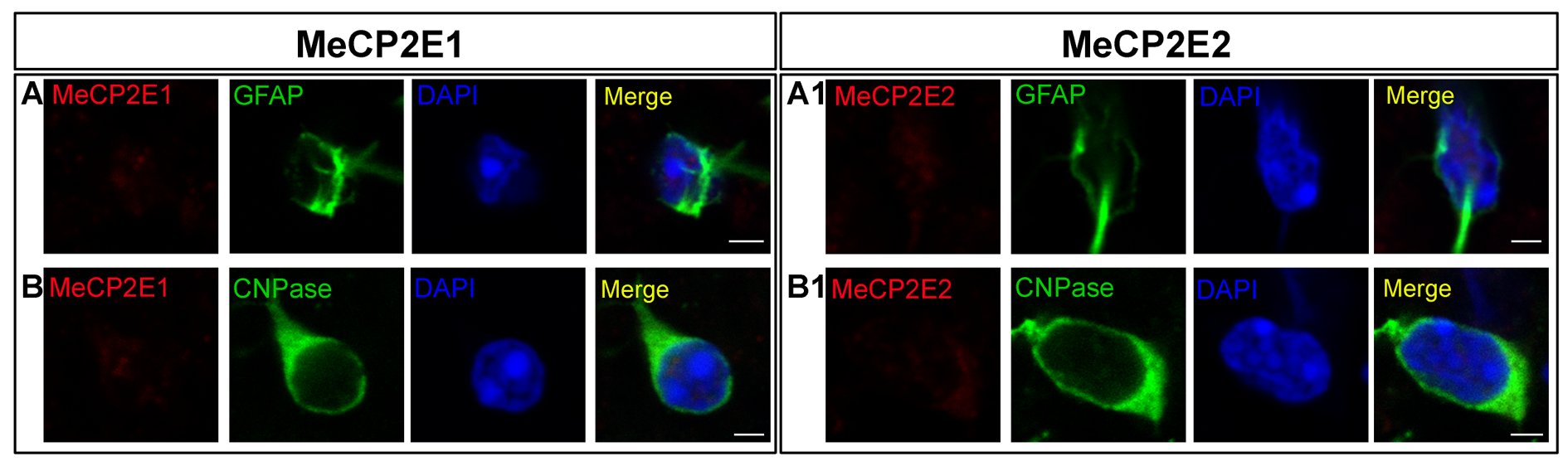

Supplement: Figure S4 — Absence of MeCP2E1- and MeCP2E2-specific signals in the GFAP+ and CNPase+ cells of the Mecp2 tm1.1Bird y/− null mouse brain hippocampus. Left panel (A-B): MeCP2E1 and, Right panel (A1-B1): MeCP2E2. Absence of the detection of MeCP2 isoforms in (A-A1) astrocytes (GFAP+), and (B-B1) oligodendrocytes (CNPase+). Scale bars represent 2 µm. All are confocal images of single nuclei. (TIF) [file pone.0090645.s004.tif]

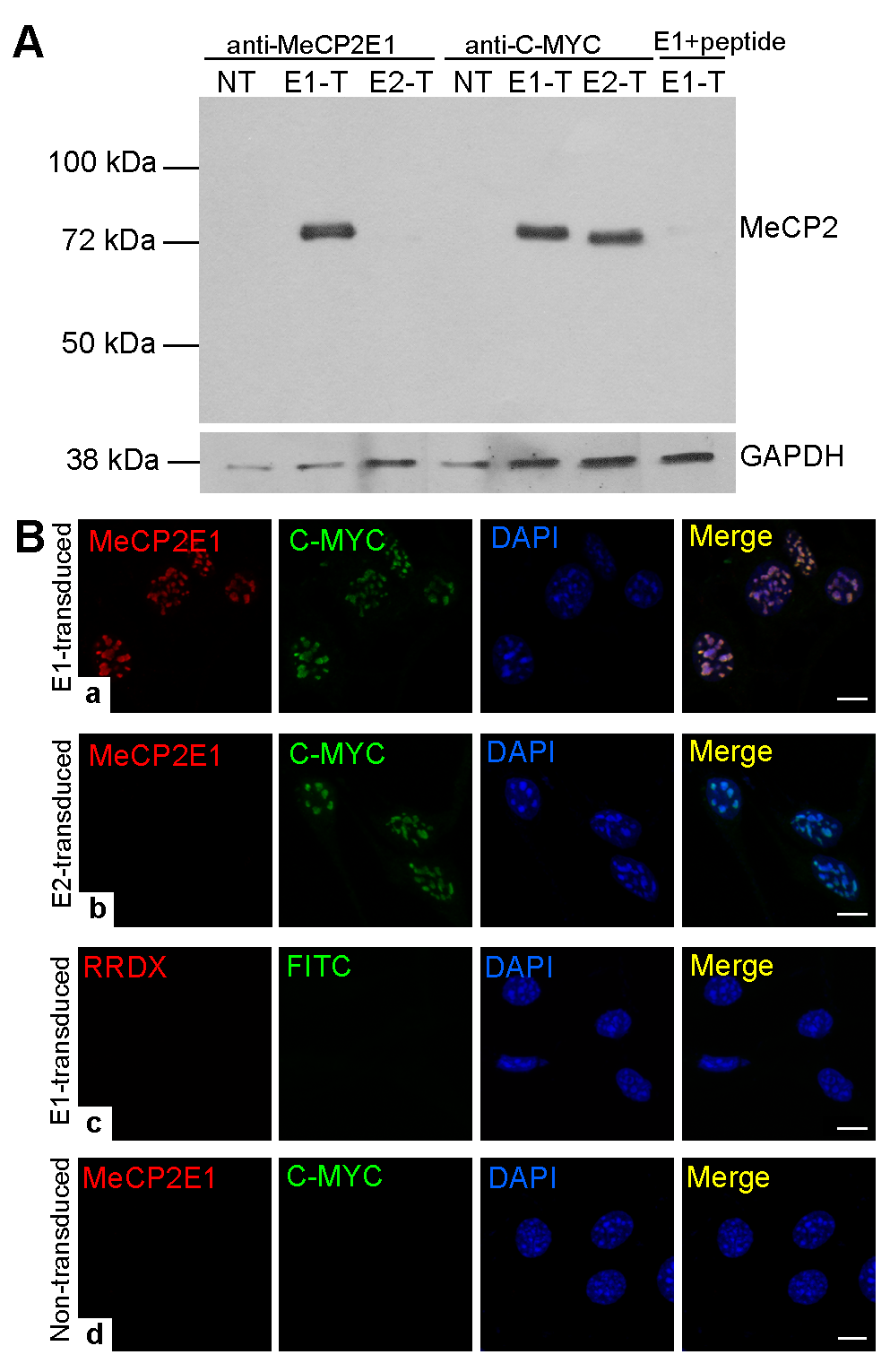

Supplement: Figure S5 — Validation of the custom-made rabbit MeCP2E1 antibody. (A) Western blot experiment to detect MeCP2E1 expression in control non-transfected (NT), MECP2E1 transfected (E1-T), MECP2E2 transfected (E2-T), and MECP2E1 pre-incubated with the antigenic peptide. Anti-MYC labelling was used as a positive control. GAPDH labelling was used as a loading control. (B) Detection of MeCP2E1 by immunofluorescence in NIH3T3 cells transduced with a) MECP2E1 or b) MECP2E2. Scale bars represent 10 µm. (TIF) [file pone.0090645.s005.tif]

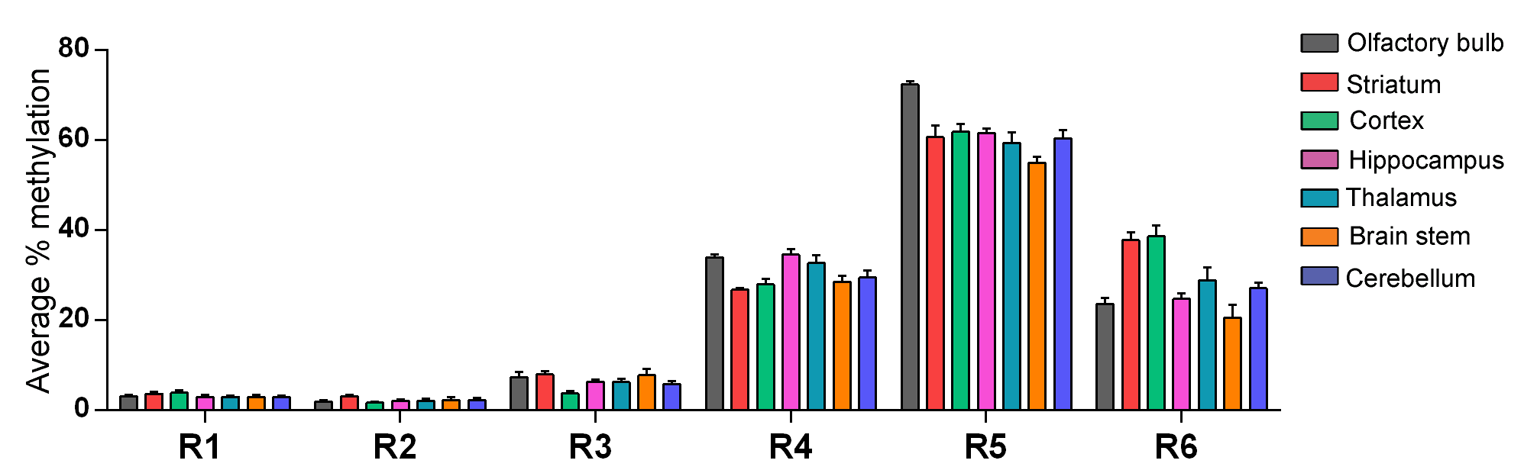

Supplement: Figure S6 — Bisulfite pyrosequencing analysis of average DNA methylation at the Mecp2 regulatory elements in adult murine brain regions. The graph represents the average percentage methylation (% Meth) observed over the entire regions in seven brain regions of the adult mouse brain. N = 5±SEM. For detailed comparison of statistical analysis, see Table S4. (TIF) [file pone.0090645.s006.tif]
