# Supplementary material for: Brain Region-Specific Expression of MeCP2 Isoforms Correlates with DNA Methylation within Mecp2 Regulatory Elements
Source: PLoS One. 2014 Mar 3;9(3):e90645. doi: 10.1371/journal.pone.0090645 (PMC3940938; doi:10.1371/journal.pone.0090645)
Supplement: Note S1 — Generation and validation of chicken polyclonal MeCP2E2 antibody. (DOCX) [file pone.0090645.s007.docx]

**Supplementary Note 1:**

***Generation and validation of chicken polyclonal MeCP2E2 antibody.***

In order to determine the endogenous expression pattern of MeCP2E2, we initially developed an anti-MeCP2E2 isoform-specific antibody using an antigenic peptide spanning a selected region of MeCP2E2 N-terminus. We validated the specificity of the anti-MeCP2E2 antibody by Western blot (WB) and immunofluorescence (IF) experiments at various stages of the antibody production and after IgY purification. For validations by WB, the affinity purified antibody was tested using protein extracts from Phoenix cells transfected with either Retro-EF1α-E1 or Retro-EF1α-E2 [[4](#_ENREF_4)] (Figure 2B), in parallel to non-transfected control cells, as previously described [[3](#_ENREF_3)]. Western blot analysis with the anti-MeCP2E2 antibody yielded a specific band at the expected molecular weight (approximately75 kDa) in *MECP2E2-*transfected cells (Figure 2C, lane 3). In contrast, no signal was detected in non-transfected cells (Figure 2C, lane 1), nor in transfected cells with *MECP2E1* (Figure 2C, lane 2). Importantly, pre-incubation of the anti-MeCP2E2 antibody with the antigenic peptide used to generate the antibody (peptide competition) eliminated the detection of signal in the *MECP2E2* transfected cells (Figure 2C, lane 7). The specificity and sensitivity of this newly developed anti-MeCP2E2 antibody was verified by pre-incubation of antibody with increasing concentrations of the antigenic peptide before probing the membranes with *MECP2E2* transfected cell lysates (Figure S2A, lanes 2–4). The presence of exogenous MeCP2 in the transfected cells with either Retro-EF1α-E1 or Retro-EF1α-E2 was verified by immunolabelling with an anti-C-MYC antibody (Figure 2C, lanes 5–6), with no detectable signal in non-transfected cells (Figure 2C, lane 4).

Further verification of the specificity of the custom-made anti-MeCP2E2 antibody using IF, revealed the localization of MeCP2E2 in the DAPI-rich heterochromatic foci within the NIH3T3 cells transduced with *MECP2E2*. No signal was detected in the *MECP2E1* transduced cells (Figure 2D; a-b). C-MYC labelling confirmed the successful transduction of both MeCP2E1 and MeCP2E2 within the tested samples (Figure 2D: a-b). The absence of endogenous *MECP2E2* expression was verified in non-transduced NIH3T3 cells using the anti-MeCP2E2 antibody (Figure S2B: a). No signal was observed in primary omission experiments using Retro-EF1α-E1 transduced cells with the same secondary antibodies (Figure S2B: b).
