# Supplementary material for: Brain Region-Specific Expression of MeCP2 Isoforms Correlates with DNA Methylation within Mecp2 Regulatory Elements
Source: PLoS One. 2014 Mar 3;9(3):e90645. doi: 10.1371/journal.pone.0090645 (PMC3940938; doi:10.1371/journal.pone.0090645)
Supplement: Note S2 — Generation and validation of rabbit polyclonal MeCP2E1 antibody. (DOCX) [file pone.0090645.s008.docx]

**Supplementary Note 2:**

***Generation and validation of rabbit polyclonal MeCP2E1 antibody.***

For double-labelling of MeCP2E1 and MeCP2E2, we required an isoform-specific antibody that is generated in a different species. Therefore, we applied the same strategy as we reported for developing anti-MeCP2E1 [[3](#_ENREF_3)], but as a rabbit polyclonal antibody. The specificity and sensitivity of this newly developed anti-MeCP2E1 antibody was initially verified by WB. Probing protein extracts from non-transfected, MeCP2E1-transfected and MeCP2E2-transfected phoenix cells, the affinity purified anti-MeCP2E1 detected specific bands at ~75 kDa in MeCP2E1-transfected extracts (Figure S5A, lane 2). No signal was detected in non-transfected cells (Figure S5A, lane 1), nor in transfected cells with *MECP2E2* (Figure S5A, lane 3). As done previously, the presence of exogenous MeCP2 in the transfected cells with either Retro-EF1α-E1 or Retro-EF1α-E2 was verified by immunolabelling with an anti-C-MYC antibody (Figure S5A, lanes 5-6), with no detectable signal in non-transfected cells (Figure S5A, lane 4). Furthermore, pre-incubation of the anti-MeCP2E1 antibody with the antigenic peptide before probing the membranes with *MECP2E1* transfected cell lysate (Figure S5A, lanes 2–5) completely abrogated the detection of exogenous MeCP2E1. IF staining with the anti-MeCP2E1 antibody revealed the expression of MeCP2 in the DAPI-rich heterochromatic foci within the NIH3T3 cells transduced with *MECP2E1*, but no signal was detected in the *MECP2E2* transduced cells (Figure S5B: a-b). This indicates that our newly developed anti-MeCP2E1 antibody does not cross-react with the overexpressed *MECP2E2*. In both *MECP2E1* and *MECP2E2* overexpressed cells, incubation with an anti-C-MYC antibody resulted in detectable signals indicating that the transduced protein is properly expressed in both cases. As expected, we did not detect any signal in primary omission experiments using Retro-EF1α-E1 transduced cells with the same secondary antibody (Figure S5B: c). The absence of endogenous *MECP2E2* expression was confirmed in the non-transduced NIH3T3 cells by using the anti-MeCP2E2 antibody (Figure S5B: d).
