# Supplementary material for: Brain Region-Specific Expression of MeCP2 Isoforms Correlates with DNA Methylation within Mecp2 Regulatory Elements
Source: PLoS One. 2014 Mar 3;9(3):e90645. doi: 10.1371/journal.pone.0090645 (PMC3940938; doi:10.1371/journal.pone.0090645)
Supplement: Table S1 — Differences of the expression of Mecp2/MeCP2 isoforms across developmental stages. (DOCX) [file pone.0090645.s009.docx]

**Table S1_as TEXT**

| **Table S1. Differences of the expression of *Mecp2*/MeCP2 isoforms across developmental stages** | | | | | | | | | | | |  |
| --- | --- | --- | --- | --- | --- | --- | --- | --- | --- | --- | --- | --- |
| **MeCP2E1** | | | **MeCP2E2** | | | ***Mecp2e1*** | | | ***Mecp2e2*** | | |  |
| **AGE** | **MD** | **SIG** | **P** | **MD** | **SIG** | **P** | **MD** | **SIG** | **P** | **MD** | **SIG** | **P** |
| E14 vs. E18 | -0.2647 | **** | < 0.0001 | -0.1089 | ns | 0.2642 | -0.00262 | ns | 0.7446 | -0.00158 | ns | 0.9979 |
| E14 vs. P1 | -0.3697 | **** | < 0.0001 | -0.4495 | **** | < 0.0001 | -0.00192 | ns | 0.9787 | -0.0051 | * | 0.0162 |
| E14 vs. P7 | -0.5155 | **** | < 0.0001 | -0.7871 | **** | < 0.0001 | 0.004596 | * | 0.0424 | -0.00472 | * | 0.0335 |
| E14 vs. P21 | -0.4578 | **** | < 0.0001 | -0.7829 | **** | < 0.0001 | 0.009475 | **** | < 0.0001 | -0.00075 | ns | > 0.9999 |
| E14 vs. P28 | -0.3498 | **** | < 0.0001 | -0.4899 | **** | < 0.0001 | 0.009832 | **** | < 0.0001 | 1.57E-05 | ns | > 0.9999 |
| E14 vs. Null | 0.5253 | **** | < 0.0001 | 0.00697 | ns | > 0.9999 | 0.01573 | **** | < 0.0001 | 0.004131 | **** | < 0.0001 |
| E18 vs. P1 | -0.105 | ns | 0.3166 | -0.3407 | **** | < 0.0001 | 0.000703 | ns | > 0.9999 | -0.00353 | * | 0.0347 |
| E18 vs. P7 | -0.2508 | **** | < 0.0001 | -0.6782 | **** | < 0.0001 | 0.007218 | *** | 0.0002 | -0.00315 | * | 0.0254 |
| E18 vs. P21 | -0.1931 | ** | 0.0016 | -0.6741 | **** | < 0.0001 | 0.0121 | **** | < 0.0001 | 0.000828 | ns | > 0.9999 |
| E18 vs. P28 | -0.0851 | ns | 0.668 | -0.3811 | **** | < 0.0001 | 0.01245 | **** | < 0.0001 | 0.001591 | ns | 0.9976 |
| E18 vs. NULL | 0.79 | **** | < 0.0001 | 0.1158 | ns | 0.1862 | 0.01835 | **** | < 0.0001 | 0.005706 | **** | < 0.0001 |
| P1 vs. P7 | -0.1458 | * | 0.0333 | -0.3375 | **** | < 0.0001 | 0.006515 | *** | 0.001 | 0.000379 | ns | > 0.9999 |
| P1 vs. P21 | -0.0881 | ns | 0.6105 | -0.3334 | **** | < 0.0001 | 0.01139 | **** | < 0.0001 | 0.004354 | *** | 0.001 |
| P1 vs. P28 | 0.0199 | ns | > 0.9999 | -0.0404 | ns | 0.9998 | 0.01175 | **** | < 0.0001 | 0.005117 | *** | 0.0005 |
| P1 vs. NULL | 0.895 | **** | < 0.0001 | 0.4565 | **** | < 0.0001 | 0.01765 | **** | < 0.0001 | 0.009232 | **** | < 0.0001 |
| P7 vs. P21 | 0.0577 | ns | 0.9837 | 0.00413 | ns | > 0.9999 | 0.004878 | * | 0.0249 | 0.003975 | ** | 0.0086 |
| P7 vs. P28 | 0.1657 | ** | 0.0096 | 0.2971 | **** | < 0.0001 | 0.005235 | * | 0.0125 | 0.004737 | ** | 0.0031 |
| P7 vs. NULL | 1.041 | **** | < 0.0001 | 0.794 | **** | < 0.0001 | 0.01113 | **** | < 0.0001 | 0.008853 | **** | < 0.0001 |
| P21 vs. P28 | 0.108 | ns | 0.2748 | 0.293 | **** | < 0.0001 | 0.000357 | ns | > 0.9999 | 0.000763 | ns | > 0.9999 |
| P21 vs. NULL | 0.9831 | **** | < 0.0001 | 0.7899 | **** | < 0.0001 | 0.006255 | *** | 0.0009 | 0.004878 | **** | < 0.0001 |
| P28 vs. NULL | 0.8751 | **** | < 0.0001 | 0.4969 | **** | < 0.0001 | 0.005898 | *** | 0.00034 | 0.004116 | **** | < 0.0001 |
| vs= versus, MD = Mean difference, SIG= Significance, P= P value  Bonferroni's multiple comparisons test. P≤0.05 was considered statistically significant. N=3 | | | | | | | | | | | | |
