# Supplementary material for: Brain Region-Specific Expression of MeCP2 Isoforms Correlates with DNA Methylation within Mecp2 Regulatory Elements
Source: PLoS One. 2014 Mar 3;9(3):e90645. doi: 10.1371/journal.pone.0090645 (PMC3940938; doi:10.1371/journal.pone.0090645)
Supplement: Table S2 — Differences of the expression of Mecp2/MeCP2 isoforms between brain regions. (DOCX) [file pone.0090645.s010.docx]

**Table S2_as TEXT**

| **Table S2. Differences of the expression of *Mecp2*/MeCP2 isoforms between brain regions** | | | | | | | | | | | |  |
| --- | --- | --- | --- | --- | --- | --- | --- | --- | --- | --- | --- | --- |
| **MeCP2E1** | | | **MeCP2E2** | | | ***Mecp2e1*** | | | ***Mecp2e2*** | | |  |
| **REGION** | **MD** | **SIG** | **P** | **MD** | **SIG** | **P** | **MD** | **SIG** | **P** | **MD** | **SIG** | **P** |
| WB vs. NULL | 0.9703 | **** | < 0.0001 | 0.3291 | **** | < 0.0001 | 0.00785 | **** | < 0.0001 | 0.002907 | * | 0.0422 |
| WB vs. OB | -0.07358 | ns | 0.997 | -0.5935 | **** | < 0.0001 | 0.001652 | ns | 0.8598 | -0.00198 | ns | 0.5525 |
| WB vs. STR | -0.08335 | ns | 0.9804 | -0.3884 | **** | < 0.0001 | -0.000085 | ns | > 0.9999 | -0.0022 | ns | 0.3449 |
| WB vs. CTX | -0.04608 | ns | > 0.9999 | -0.1679 | ns | 0.0638 | 0.000652 | ns | > 0.9999 | -0.00397 | *** | 0.001 |
| WB vs. HIPPO | -0.03852 | ns | > 0.9999 | -0.3517 | **** | < 0.0001 | 0.001999 | ns | 0.5294 | -0.00112 | ns | 0.9993 |
| WB vs. THAL | -0.05912 | ns | > 0.9999 | -0.0257 | ns | > 0.9999 | -0.00083 | ns | > 0.9999 | -0.00084 | ns | > 0.9999 |
| WB vs. BS | -0.08505 | ns | 0.9745 | 0.1816 | * | 0.0302 | 0.001533 | ns | 0.9312 | 0.00059 | ns | > 0.9999 |
| WB vs. CERE | -0.07208 | ns | 0.9979 | -0.6069 | **** | < 0.0001 | 0.001866 | ns | 0.6671 | -0.00212 | ns | 0.4079 |
| OB vs. STR | -0.00977 | ns | > 0.9999 | 0.2051 | ** | 0.0078 | -0.001738 | ns | 0.7902 | -0.00022 | ns | > 0.9999 |
| OB vs. CTX | 0.0275 | ns | > 0.9999 | 0.4256 | **** | < 0.0001 | -0.001 | ns | > 0.9999 | -0.00199 | ns | 0.5356 |
| OB vs. HIPPO | 0.03507 | ns | > 0.9999 | 0.2418 | *** | 0.0009 | 0.00034 | ns | > 0.9999 | 0.000854 | ns | > 0.9999 |
| OB vs. THAL | 0.01447 | ns | > 0.9999 | 0.5678 | **** | < 0.0001 | -0.002482 | ns | 0.1596 | 0.001139 | ns | 0.9991 |
| OB vs. BS | -0.01147 | ns | > 0.9999 | 0.7751 | **** | < 0.0001 | -0.00011 | ns | > 0.9999 | 0.002566 | ns | 0.1244 |
| OB vs. CERE | 0.0015 | ns | > 0.9999 | -0.0134 | ns | > 0.9999 | 0.000213 | ns | > 0.9999 | -0.00015 | ns | > 0.9999 |
| STR vs. CTX | 0.03727 | ns | > 0.9999 | 0.2205 | ** | 0.0031 | 0.000737 | ns | > 0.9999 | -0.00177 | ns | 0.7573 |
| STR vs. HIPPO | 0.04483 | ns | > 0.9999 | 0.0367 | ns | > 0.9999 | 0.002085 | ns | 0.4441 | 0.001073 | ns | 0.9997 |
| STR vs. THAL | 0.02423 | ns | > 0.9999 | 0.3627 | **** | < 0.0001 | -0.000744 | ns | > 0.9999 | 0.001358 | ns | 0.9843 |
| STR vs. BS | -0.0017 | ns | > 0.9999 | 0.57 | **** | < 0.0001 | 0.001619 | ns | 0.8827 | 0.002785 | ns | 0.0628 |
| STR vs. CERE | 0.01127 | ns | > 0.9999 | -0.2185 | ** | 0.0035 | 0.001951 | ns | 0.5783 | 7.19E-05 | ns | > 0.9999 |
| CTX vs. HIPPO | 0.00756 | ns | > 0.9999 | -0.1838 | * | 0.0267 | 0.001347 | ns | 0.986 | 0.002847 | ns | 0.0514 |
| CTX vs. THAL | -0.01303 | ns | > 0.9999 | 0.1422 | ns | 0.2286 | -0.001482 | ns | 0.9526 | 0.003132 | * | 0.0197 |
| CTX vs. BS | -0.03897 | ns | > 0.9999 | 0.3495 | **** | < 0.0001 | 0.000881 | ns | > 0.9999 | 0.004559 | *** | 0.0008 |
| CTX vs. CERE | -0.026 | ns | > 0.9999 | -0.439 | **** | < 0.0001 | 0.001214 | ns | 0.9972 | 0.001846 | ns | 0.6871 |
| HIPPO vs. THAL | -0.0206 | ns | > 0.9999 | 0.326 | **** | < 0.0001 | -0.002829 | ns | 0.0544 | 0.000285 | ns | > 0.9999 |
| HIPPO vs. BS | -0.04653 | ns | > 0.9999 | 0.5333 | **** | < 0.0001 | -0.000465 | ns | > 0.9999 | 0.001713 | ns | 0.8122 |
| HIPPO vs. CERE | -0.03357 | ns | > 0.9999 | -0.2552 | *** | 0.0004 | -0.000133 | ns | > 0.9999 | -0.001 | ns | > 0.9999 |
| THAL vs. BS | -0.02593 | ns | > 0.9999 | 0.2073 | ** | 0.0069 | 0.002363 | ns | 0.2233 | 0.001427 | ns | 0.9698 |
| THAL vs. CERE | -0.01297 | ns | > 0.9999 | -0.5812 | **** | < 0.0001 | 0.002696 | ns | 0.0835 | -0.00129 | ns | 0.9929 |
| BS vs. CERE | 0.01297 | ns | > 0.9999 | -0.7885 | **** | < 0.0001 | 0.000332 | ns | > 0.9999 | -0.00271 | ns | 0.079 |
| vs= versus; MD = Mean difference; SIG= Significance; P= P value  Bonferroni's multiple comparisons test. P≤0.05 was considered statistically significant. N=3 | | | | | | | | | | | | |
