# Supplementary material for: Brain Region-Specific Expression of MeCP2 Isoforms Correlates with DNA Methylation within Mecp2 Regulatory Elements
Source: PLoS One. 2014 Mar 3;9(3):e90645. doi: 10.1371/journal.pone.0090645 (PMC3940938; doi:10.1371/journal.pone.0090645)
Supplement: Table S3 — Comparison of percentage methylation differences at the individual CpG sites between brain regions. (DOCX) [file pone.0090645.s011.docx]

**Table S3_as TEXT**

| **Table S3. Comparison of percentage methylation differences at the individual CpG sites between brain regions** | | | | | | | | | | | | | | |  |
| --- | --- | --- | --- | --- | --- | --- | --- | --- | --- | --- | --- | --- | --- | --- | --- |
| **R1 CpG 5** | | | **R3 CpG 2** | | | **R5 CpG 1** | | | **R6 CpG 1** | | | **R6 CpG 2** | | |  |
| **REGION** | **MD** | **SIG** | **P** | **MD** | **SIG** | **P** | **MD** | **SIG** | **P** | **MD** | **SIG** | **P** | **MD** | **SIG** | **P** |
| OB vs. STR | -1.273 | ns | > 0.9999 | -0.5617 | ns | > 0.9999 | 11.67 | *** | 0.0005 | -22.93 | **** | < 0.0001 | -5.306 | ns | 0.3046 |
| OB vs. CTX | -3.336 | *** | 0.0009 | 8.247 | **** | < 0.0001 | 10.49 | ** | 0.0024 | -19.51 | **** | < 0.0001 | -10.55 | ** | 0.0020 |
| OB vs. HIPPO | -0.08033 | ns | > 0.9999 | 1.768 | ns | 0.6119 | 10.73 | ** | 0.0019 | -1.456 | ns | 0.7941 | -0.8000 | ns | 0.9945 |
| OB vs. THAL | -0.1658 | ns | > 0.9999 | 2.523 | * | 0.0400 | 12.94 | **** | < 0.0001 | -10.59 | ** | 0.0015 | 0.1360 | ns | 0.9945 |
| OB vs. BS | -0.3343 | ns | > 0.9999 | -0.7775 | ns | > 0.9999 | 17.36 | **** | < 0.0001 | -3.076 | ns | 0.5986 | 9.134 | ** | 0.0098 |
| OB vs. CERE | -0.4003 | ns | > 0.9999 | 2.840 | * | 0.0101 | 11.90 | *** | 0.0004 | 4.424 | ns | 0.4133 | -11.39 | *** | 0.0007 |
| STR vs. CTX | -2.063 | ns | 0.2311 | 8.809 | **** | < 0.0001 | -1.178 | ns | 0.9993 | 3.420 | ns | 0.5986 | -5.245 | ns | 0.3046 |
| STR vs. HIPPO | 1.193 | ns | > 0.9999 | 2.330 | ns | 0.0862 | -0.9420 | ns | 0.9993 | 21.48 | **** | < 0.0001 | 4.506 | ns | 0.3931 |
| STR vs. THAL | 1.108 | ns | > 0.9999 | 3.084 | ** | 0.0032 | 1.274 | ns | 0.9993 | 12.34 | *** | 0.0001 | 5.442 | ns | 0.3046 |
| STR vs. BS | 0.9390 | ns | > 0.9999 | -0.2158 | ns | > 0.9999 | 5.694 | ns | 0.3756 | 19.86 | **** | < 0.0001 | 14.44 | **** | < 0.0001 |
| STR vs. CERE | 0.8730 | ns | > 0.9999 | 3.402 | *** | 0.0006 | 0.2310 | ns | 0.9993 | 27.36 | **** | < 0.0001 | -6.086 | ns | 0.2029 |
| CTX vs. HIPPO | 3.256 | ** | 0.0014 | -6.479 | **** | < 0.0001 | 0.2360 | ns | 0.9993 | 18.06 | **** | < 0.0001 | 9.751 | ** | 0.0049 |
| CTX vs. THAL | 3.170 | ** | 0.0021 | -5.725 | **** | < 0.0001 | 2.452 | ns | 0.9888 | 8.921 | * | 0.0104 | 10.69 | ** | 0.0019 |
| CTX vs. BS | 3.002 | ** | 0.0048 | -9.025 | **** | < 0.0001 | 6.872 | ns | 0.1601 | 16.44 | **** | < 0.0001 | 19.69 | **** | < 0.0001 |
| CTX vs. CERE | 2.936 | ** | 0.0065 | -5.408 | **** | < 0.0001 | 1.409 | ns | 0.9993 | 23.94 | **** | < 0.0001 | -0.8405 | ns | 0.9945 |
| HIPPO vs. THAL | -0.0855 | ns | > 0.9999 | 0.7542 | ns | > 0.9999 | 2.216 | ns | 0.9913 | -9.136 | ** | 0.0089 | 0.9360 | ns | 0.9945 |
| HIPPO vs. BS | -0.2540 | ns | > 0.9999 | -2.546 | * | 0.0363 | 6.636 | ns | 0.1867 | -1.620 | ns | 0.7941 | 9.934 | ** | 0.0042 |
| HIPPO vs. CERE | -0.3200 | ns | > 0.9999 | 1.072 | ns | > 0.9999 | 1.173 | ns | 0.9993 | 5.880 | ns | 0.1675 | -10.59 | ** | 0.0020 |
| THAL vs. BS | -0.1685 | ns | > 0.9999 | -3.300 | ** | 0.0011 | 4.420 | ns | 0.6918 | 7.516 | * | 0.0461 | 8.998 | * | 0.0106 |
| THAL vs. CERE | -0.2345 | ns | > 0.9999 | 0.3175 | ns | > 0.9999 | -1.043 | ns | 0.9993 | 15.02 | **** | < 0.0001 | -11.53 | *** | 0.0006 |
| BS vs. CERE | -0.06600 | ns | > 0.9999 | 3.618 | *** | 0.0002 | -5.463 | ns | 0.4143 | 7.500 | * | 0.0461 | -20.53 | **** | < 0.0001 |
| vs= versus; MD = Mean difference; SIG= Significance; P= P value  Bonferroni's multiple comparisons test. P≤0.05 was considered statistically significant. N=5 | | | | | | | | | | | | | | | |
