# Supplementary material for: Brain Region-Specific Expression of MeCP2 Isoforms Correlates with DNA Methylation within Mecp2 Regulatory Elements
Source: PLoS One. 2014 Mar 3;9(3):e90645. doi: 10.1371/journal.pone.0090645 (PMC3940938; doi:10.1371/journal.pone.0090645)
Supplement: Table S4 — Comparison of average percentage methylation differences between brain regions. (DOCX) [file pone.0090645.s012.docx]

**Table S4_as TEXT**

|  | **Table S4. Comparison of average percentage methylation differences between brain regions** | | |
| --- | --- | --- | --- |
|  | **R6 Av** | | |
| **REGIONS** | **MD** | **SIG** | **P** |
| OB vs. STR | -14.12 | **** | < 0.0001 |
| OB vs. CTX | -15.03 | **** | < 0.0001 |
| OB vs. HIPPO | -1.128 | ns | > 0.9999 |
| OB vs. THAL | -5.228 | ns | 0.0985 |
| OB vs. BS | 3.029 | ns | > 0.9999 |
| OB vs. CERE | -3.484 | ns | > 0.9999 |
| STR vs. CTX | -0.9130 | ns | > 0.9999 |
| STR vs. HIPPO | 12.99 | **** | < 0.0001 |
| STR vs. THAL | 8.891 | **** | < 0.0001 |
| STR vs. BS | 17.15 | **** | < 0.0001 |
| STR vs. CERE | 10.64 | **** | < 0.0001 |
| CTX vs. HIPPO | 13.90 | **** | < 0.0001 |
| CTX vs. THAL | 9.804 | **** | < 0.0001 |
| CTX vs. BS | 18.06 | **** | < 0.0001 |
| CTX vs. CERE | 11.55 | **** | < 0.0001 |
| HIPPO vs. THAL | -4.100 | ns | 0.5439 |
| HIPPO vs. BS | 4.157 | ns | 0.5026 |
| HIPPO vs. CERE | -2.356 | ns | > 0.9999 |
| THAL vs. BS | 8.257 | *** | 0.0002 |
| THAL vs. CERE | 1.744 | ns | > 0.9999 |
| BS vs. CERE | -6.513 | ** | 0.0098 |
| vs= versus; MD = Mean difference; SIG= Significance; P= P value  Bonferroni's multiple comparisons test. P≤0.05 was considered statistically significant. The statistically significant differences are shaded. N=5 | | | |
