# Supplementary material for: Brain Region-Specific Expression of MeCP2 Isoforms Correlates with DNA Methylation within Mecp2 Regulatory Elements
Source: PLoS One. 2014 Mar 3;9(3):e90645. doi: 10.1371/journal.pone.0090645 (PMC3940938; doi:10.1371/journal.pone.0090645)
Supplement: Table S5 — List of primers used in qRT-PCR. (DOCX) [file pone.0090645.s013.docx]

**Table S5_as TEXT**

| **Table S5. List of primers used in qRT-PCR** | | | |
| --- | --- | --- | --- |
| **Gene** | **Direction** | **Sequence (5’ to 3’)** | **Reference** |
| ***Mecp2e1*** | Forward | AGG AGA GAC TGG AGG AAA AGT | [[1](#_ENREF_1)] |
|  | Reverse | CTT AAA CTT CAG TGG CTT GTC TCT G |  |
| ***Mecp2e2*** | Forward | CTC ACC AGT TCC TGC TTT GAT GT |  |
|  | Reverse | CTT AAA CTT CAG TGG CTT GTC TCT G |  |
| ***Gapdh*** | Forward | AAC GAC CCC TTC ATT GAC | [[1](#_ENREF_1),[2](#_ENREF_2)] |
|  | Reverse | TCC ACG ACA TAC TCA GCA C |  |
