# Supplementary material for: Brain Region-Specific Expression of MeCP2 Isoforms Correlates with DNA Methylation within Mecp2 Regulatory Elements
Source: PLoS One. 2014 Mar 3;9(3):e90645. doi: 10.1371/journal.pone.0090645 (PMC3940938; doi:10.1371/journal.pone.0090645)
Supplement: Table S6 — Primary Antibodies. (DOCX) [file pone.0090645.s014.docx]

**Table S6_as TEXT**

| **Table S6. Primary Antibodies** | | | |
| --- | --- | --- | --- |
| **Primary Antibody** | **Application** | **Description** | **Source** |
| MeCP2 (C-terminal) | IHC 1:300 | Rabbit polyclonal | Millipore, 07-013 |
| MeCP2 (C-terminal) | WB 1:100 | Mouse monoclonal | Abcam, Ab50005 |
| MeCP2E1 | WB 1:100, IHC: 3mg/ml | Chicken polyclonal | Custom-made [[3](#_ENREF_3)] |
| MeCP2E2 | WB 1:100, IF 1:200,  IHC: 1mg/ml | Chicken polyclonal | Custom-made |
| MeCP2E1 | WB 1:1000,  IHC: 3mg/ml | Rabbit polyclonal | Custom-made |
| GAPDH | WB 1:500 | Rabbit polyclonal | Santa Cruz, Sc 25778 |
| Beta-ACTIN | WB 1:2000 | Mouse monoclonal | Sigma Aldrich, A2228 |
| C-MYC | WB 1:1500, IF 1:200 | Rabbit polyclonal | Santa Cruz, Sc789 |
| C-MYC | IF 1:200 | Mouse monoclonal | Invitrogen, 21280 |
| GFAP | IHC 1:500 | Mouse monoclonal | Invitrogen, 421262 |
| NEUN | IHC 1:400 | Mouse monoclonal | Millipore, Mab377 |
| CNPase | IHC 1:5000 | Mouse monoclonal | Covance, SMI-91R |
