# Supplementary material for: Brain Region-Specific Expression of MeCP2 Isoforms Correlates with DNA Methylation within Mecp2 Regulatory Elements
Source: PLoS One. 2014 Mar 3;9(3):e90645. doi: 10.1371/journal.pone.0090645 (PMC3940938; doi:10.1371/journal.pone.0090645)
Supplement: Table S7 — Secondary antibodies. (DOCX) [file pone.0090645.s015.docx]

**Table S7_as TEXT**

| **Table S7. Secondary antibodies** | | |
| --- | --- | --- |
| **Secondary Antibody** | **Application and dilution** | **Source** |
| Rhodamine Red-X conjugated goat anti mouse IgG | IF 1:400 | Jackson Immunoresearch, 115-259-146 |
| Dylight 649 conjugated goat anti chicken IgY | IF 1:400 | Jackson Immunoresearch, 103-485-155 |
| FITC-Conjugated Affinipure goat anti rabbit IgG | IF 1:400 | Jackson Immunoresearch, 111-095-144 |
| Rhodamine Red-X conjugated goat anti chicken IgY | IHC 1:400 | Jackson Immunoresearch, 103-295-155 |
| Alexa 488 goat anti rabbit | IHC 1:1000 | Invitrogen, A11034 |
| Alexa 488 goat anti mouse | IHC 1:1000 | Invitrogen, A11017 |
| Alexa 488 goat anti chicken | IHC 1:1000 | Invitrogen, A11042 |
| Peroxidase-Affinipure sheep anti-mouse IgG | WB 1:7500 | Jackson ImmunoResearch 115-035-174 |
| Peroxidase-AffiniPure donkey anti-rabbit IgG | WB 1:7500 | Jackson ImmunoResearch 711-036-152 |
| Peroxidase-AffiniPure goat anti-chicken IgY | WB 1:5000 | Jackson ImmunoResearch 103-035-155 |
