# Supplementary material for: Brain Region-Specific Expression of MeCP2 Isoforms Correlates with DNA Methylation within Mecp2 Regulatory Elements
Source: PLoS One. 2014 Mar 3;9(3):e90645. doi: 10.1371/journal.pone.0090645 (PMC3940938; doi:10.1371/journal.pone.0090645)
Supplement: Table S8 — List of primers used in bisulfite pyrosequencin. (DOCX) [file pone.0090645.s016.docx]

**Table S8_as TEXT**

| **Table S8. List of primers used in bisulfite pyrosequencing** | | |
| --- | --- | --- |
| ***Mecp2* region** | **Sequence** | **References** |
| ***Region 1*** | F1: 5’-TGGGTTTTATAATTAATGAAGGGTAA-3’ | [[1](#_ENREF_1)] |
|  | R1: 5’- CGCCAGGGTTTTCCCAGTCACGACATTTTACCACAACCCTCTCT-3’ |  |
|  | S1: 5’-AGGTGTAGTAGTATATAGG-3’ |  |
| ***Region 2*** | F1: 5’-AGTTTGGGTTTTATAATTAATGAAGGG-3’ |  |
|  | R1: 5’- CGCCAGGGTTTTCCCAGTCACGACATTTTACCACAACCCTCTCT-3’ |  |
|  | S1: 5’-AAGGGTAATTTAGATAAAGAGTAAG-3’ |  |
| ***Region 3*** | F1: 5’- GGTGAATTATTTAGTAGGGAGGTTTTAA -3’ |  |
|  | R1: 5’- CGCCAGGGTTTTCCCAGTCACGACAAAAAAAAAACCAACCCCATTCAACTAC -3’ |  |
|  | S1: 5’- AGTAGGGAGGTTTTAATAG -3’ |  |
| ***Region 4*** | F1: 5’- GTTTTAAAAAGTTTTGGGAAAAGGTGTAGT -3’ |  |
|  | R1: 5’- CGCCAGGGTTTTCCCAGTCACGACCTAAACCCTAACATCCCAACTACCAT-3’ |  |
|  | S1: 5’- AGTTTAATGGGGATTTTTAATT -3’ |  |
| ***Region 5*** | F1: 5’- AGTAGAAGTTATTATTTGTGGTGTGTAT -3’ |  |
|  | R1: 5’- CGCCAGGGTTTTCCCAGTCACGACACTATATTACTTCCCAACTCAACTAATT -3’ |  |
|  | S1: 5’- AGAGGTGTAAGGATTTT -3’ |  |
| ***Region 6*** | F1: 5’- GAAGTAGGAAGAATTGAGTTTGAGGATAG -3’ |  |
|  | R1: 5’- CGCCAGGGTTTTCCCAGTCACGACATCTATACACTACCCACATATAATACC -3’ |  |
|  | S1: 5’- GTTTGAGGATAGTTTGAAT -3’ |  |
| F: Forward PCR primer, R: Reverse PCR primer (Biotinylated), S: Sequencing primer | |  |
