# Supplementary figures and images for: Correction: Brain Region-Specific Expression of MeCP2 Isoforms Correlates with DNA Methylation within Mecp2 Regulatory Elements
Source: PLoS One. 2014 Jul 1;9(7):e101030. doi: 10.1371/journal.pone.0101030 (PMC4077763; doi:10.1371/journal.pone.0101030)

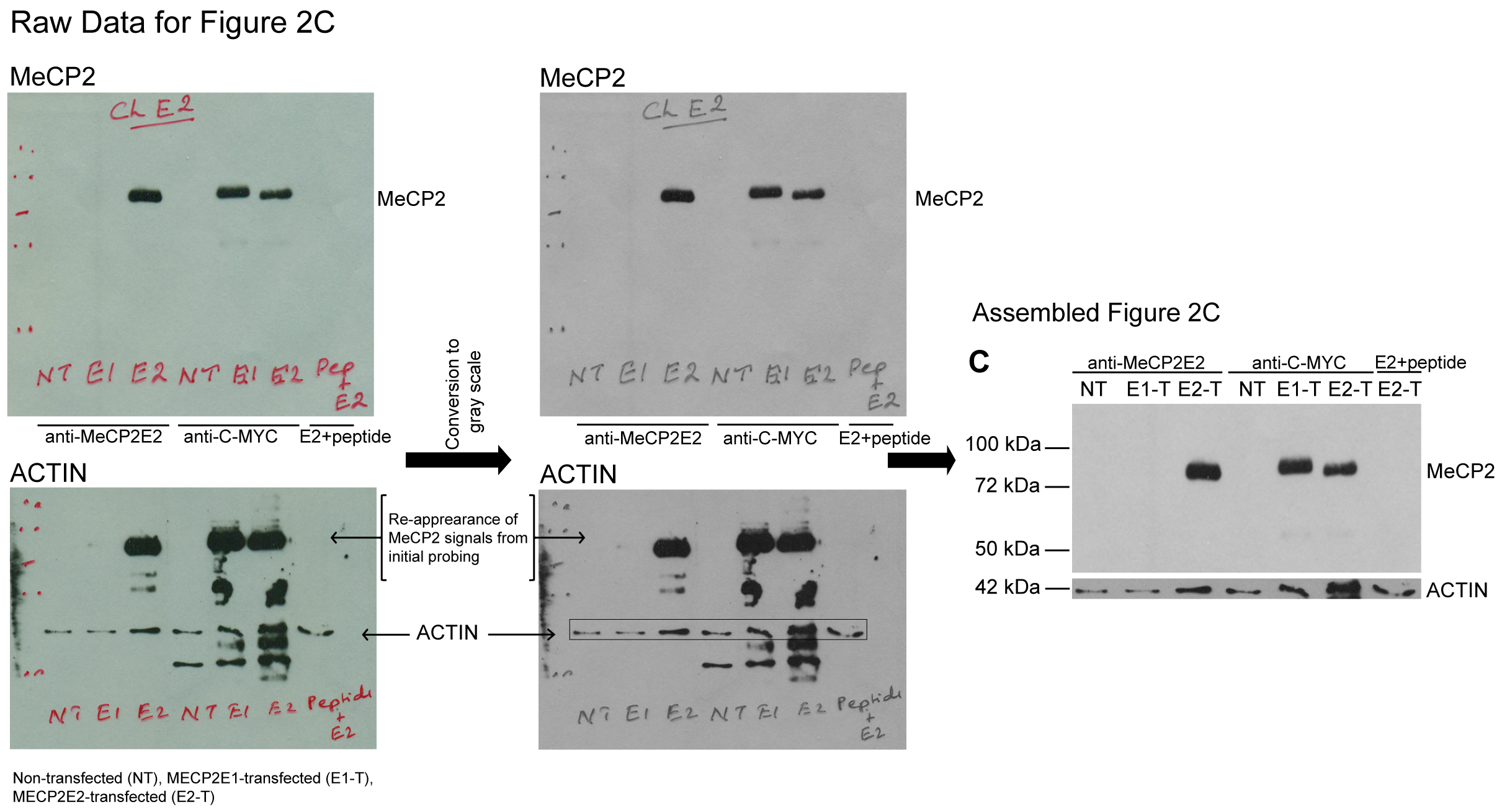

Supplement: File S1. Raw Data for Figure 2C [file pone.0101030.s001.tif]

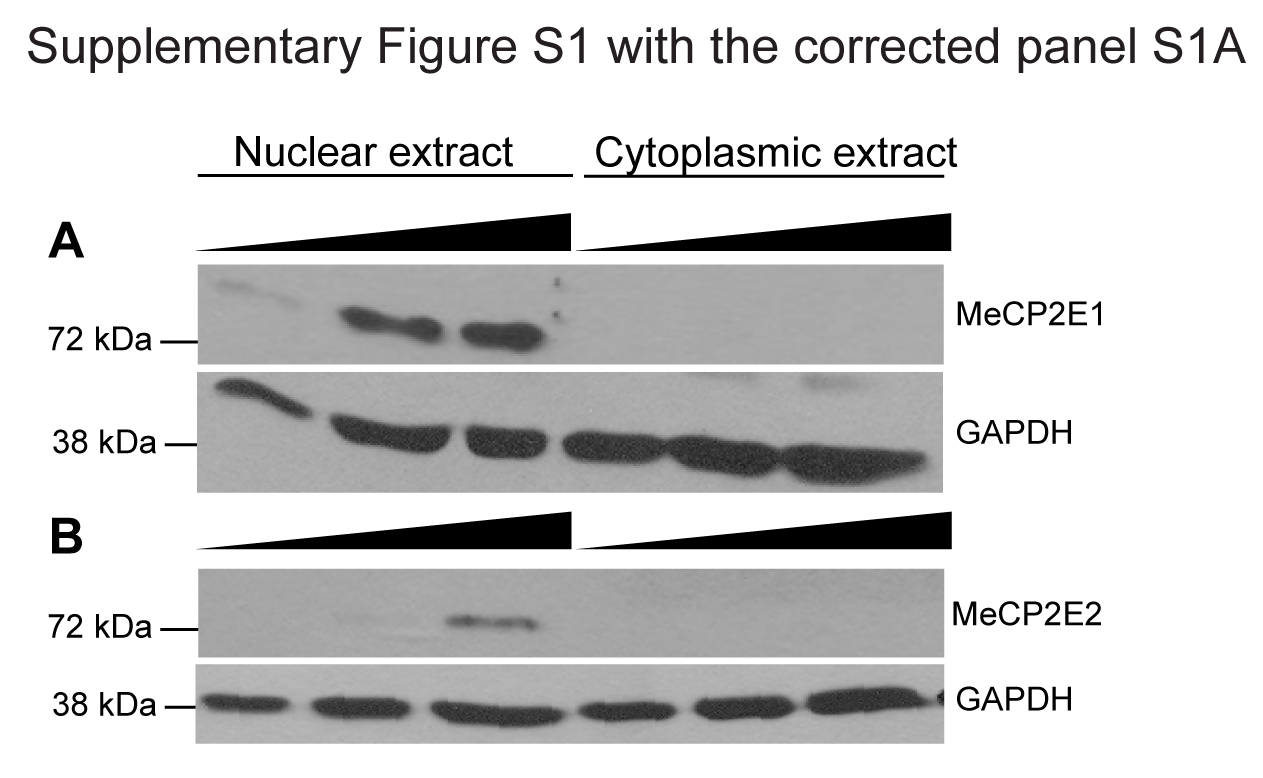

Supplement: Figure S1. Supplementary Figure S1 with the corrected panel S1A [file pone.0101030.s002.tif]

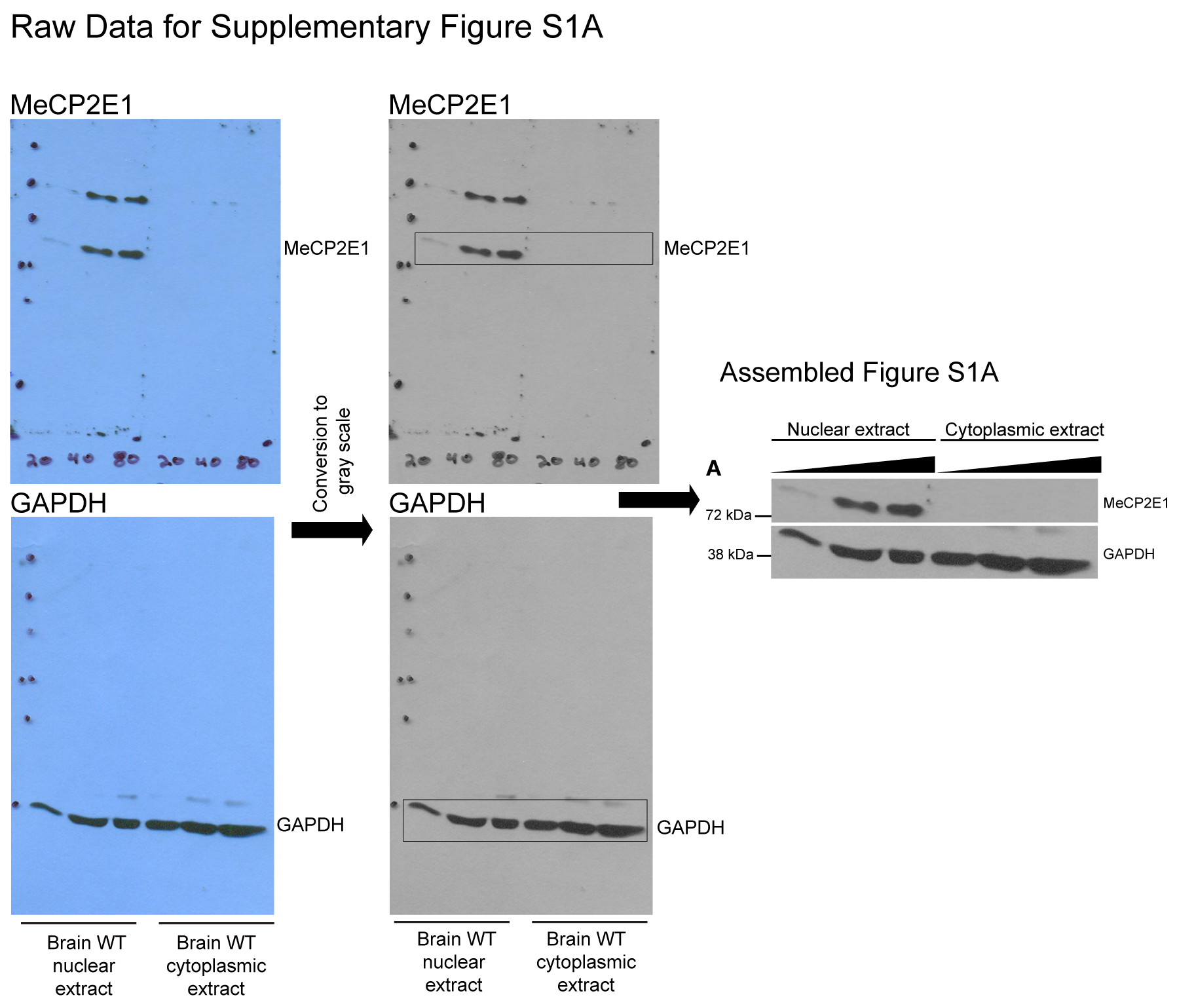

Supplement: File S2. Raw Data for Supplementary Figure S1A [file pone.0101030.s003.tif]

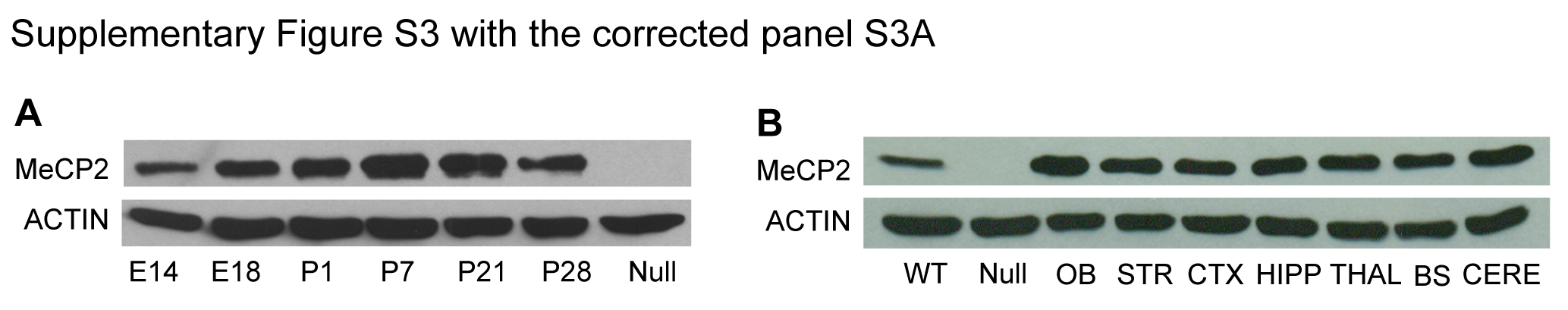

Supplement: Figure S3. Supplementary Figure S3 with the corrected panel S3A [file pone.0101030.s004.tif]

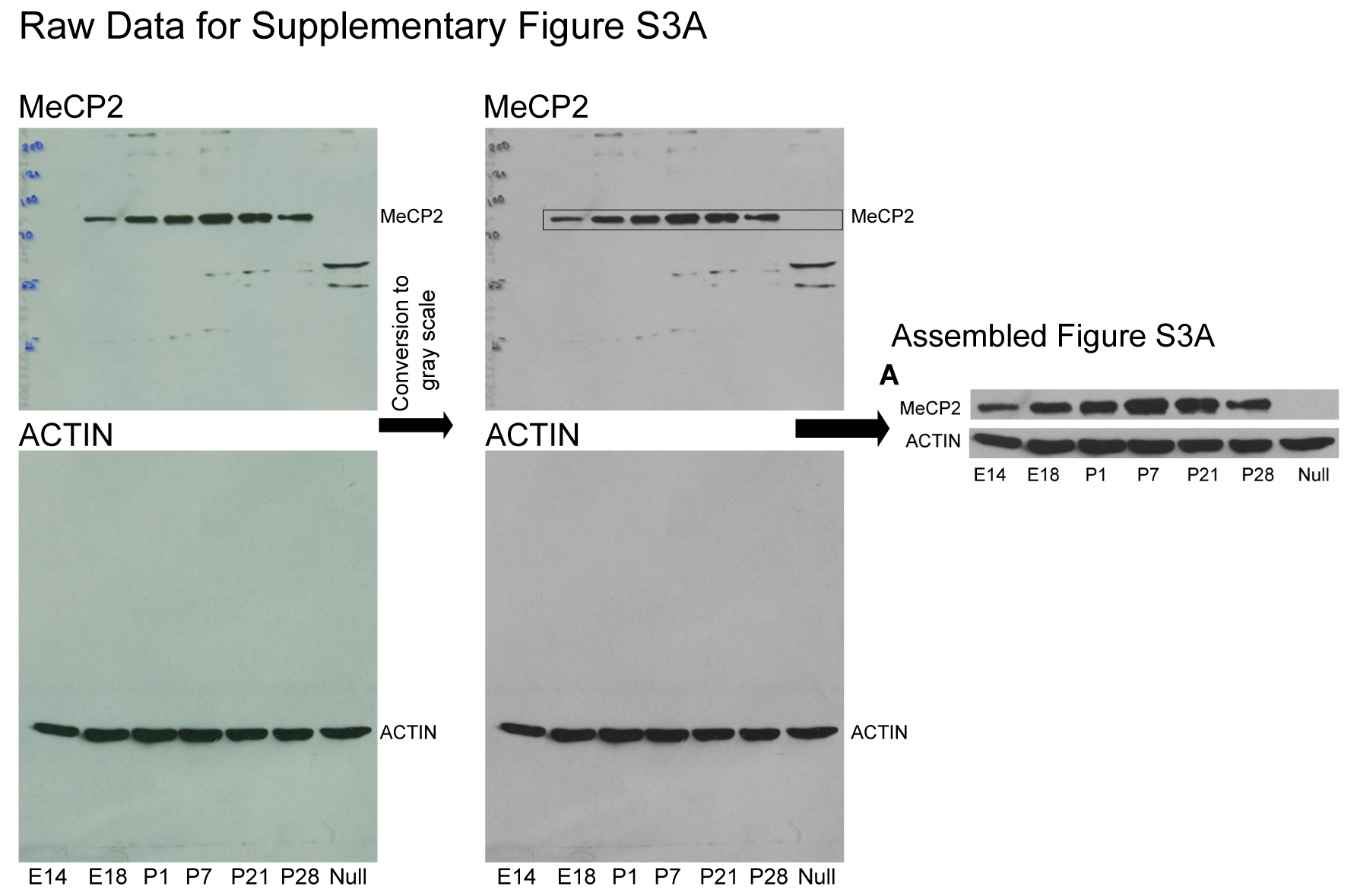

Supplement: File S3. Raw Data for Supplementary Figure S3A [file pone.0101030.s005.tif]
